# A Joint Venture of Ab Initio Molecular Dynamics, Coupled Cluster Electronic Structure Methods, and Liquid-State Theory to Compute Accurate Isotropic Hyperfine Constants of Nitroxide Probes in Water

**DOI:** 10.1021/acs.jctc.1c00582

**Published:** 2021-09-13

**Authors:** Bikramjit Sharma, Van Anh Tran, Tim Pongratz, Laura Galazzo, Irina Zhurko, Enrica Bordignon, Stefan M. Kast, Frank Neese, Dominik Marx

**Affiliations:** † Lehrstuhl für Theoretische Chemie, Ruhr-Universität Bochum, 44780 Bochum, Germany; ‡ Max-Planck-Institut für Kohlenforschung, Kaiser-Wilhelm-Platz 1, 45470 Mülheim an der Ruhr, Germany; § Physikalische Chemie III, Technische Universität Dortmund, Otto-Hahn-Str. 4a, 44227 Dortmund, Germany; ∥ Faculty of Chemistry and Biochemistry, Ruhr University Bochum, 44780 Bochum, Germany; ⊥ Laboratory of Nitrogen Compounds, N.N. Vorozhtsov Novosibirsk Institute of Organic Chemistry, NIOCH SB RAS, 9 Lavrentiev Avenue, 630090, Novosibirsk, Russia

## Abstract

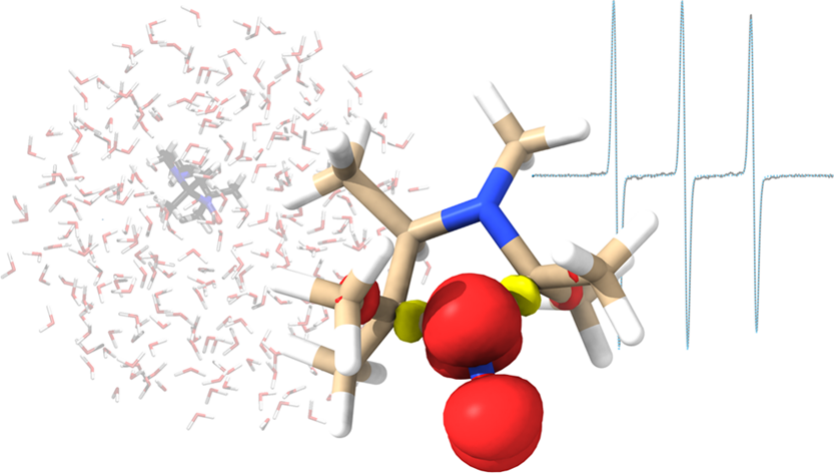

The isotropic hyperfine coupling constant (HFCC, *A*^iso^) of a pH-sensitive spin probe in a solution, HMI (2,2,3,4,5,5-hexamethylimidazolidin-1-oxyl, C_9_H_19_N_2_O) in water, is computed using an ensemble of state-of-the-art computational techniques and is gauged against X-band continuous wave electron paramagnetic resonance (EPR) measurement spectra at room temperature. Fundamentally, the investigation aims to delineate the cutting edge of current first-principles-based calculations of EPR parameters in aqueous solutions based on using rigorous statistical mechanics combined with correlated electronic structure techniques. In particular, the impact of solvation is described by exploiting fully atomistic, RISM integral equation, and implicit solvation approaches as offered by ab initio molecular dynamics (AIMD) of the periodic bulk solution (using the spin-polarized revPBE0-D3 hybrid functional), embedded cluster reference interaction site model integral equation theory (EC-RISM), and polarizable continuum embedding (using CPCM) of microsolvated complexes, respectively. HFCCs are obtained from efficient coupled cluster calculations (using open-shell DLPNO-CCSD theory) as well as from hybrid density functional theory (using revPBE0-D3). Re-solvation of “vertically desolvated” spin probe configuration snapshots by EC-RISM embedding is shown to provide significantly improved results compared to CPCM since only the former captures the inherent structural heterogeneity of the solvent close to the spin probe. The average values of the *A*^iso^ parameter obtained based on configurational statistics using explicit water within AIMD and from EC-RISM solvation are found to be satisfactorily close. Using either such explicit or RISM solvation in conjunction with DLPNO-CCSD calculations of the HFCCs provides an average *A*^iso^ parameter for HMI in aqueous solution at 300 K and 1 bar that is in good agreement with the experimentally determined one. The developed computational strategy is general in the sense that it can be readily applied to other spin probes of similar molecular complexity, to aqueous solutions beyond ambient conditions, as well as to other solvents in the longer run.

## 1 Introduction

Electron paramagnetic resonance (EPR) spectroscopy is a robust analysis tool which has found a myriad of applications across many disciplines.^1−7^ The microscopic understanding of experimental EPR signals is necessary to infer structural information of systems under investigation. EPR experiments in aqueous solutions are particularly interesting as hydration plays an important role in a wealth of diverse phenomena involving species with unpaired electrons.^7−9^ Thus, it is important to understand the link between microscopic solvation and EPR signals at the molecular level. Fundamentally, theoretical methods can dig out what is hidden behind the numbers obtained from experiments that reflect an average of a distribution of microscopic states. Obviously, an aqueous solution is dynamic as the solute and the solvent are in constant motion and during the course of their respective trajectories intermittently form hydrogen bonds or interact via other intermolecular forces. The experimental spectra reflect the long-time average of the entire ensemble and hence only see an “average picture” of the system under investigation. However, careful theoretical studies can provide detailed insights into the microscopic behavior of the solute interacting with the solvent, provided that it has been demonstrated that the theoretical results are consistent with the experimental observations. The first step of predictive theoretical investigations of any experimental result is to calculate the experimental observables as accurately as possible. In the case of EPR spectroscopy, perhaps the most important experimental observable is the hyperfine coupling constant (HFCC) that depends on the specific nucleus under consideration. For EPR probes in solution at ambient temperature, where the environment is isotropic on average, the isotropic HFCC (*A*^iso^) is one of the most important EPR observables in practice.

Computing the EPR parameter of spin probes, in particular, HFCCs, has a long and thus rich history in the literature. A host of different electronic structure methods have been used to compute such parameters based on static structures being mostly optimized equilibrium structures of the open-shell species under vacuum.^10−25^ These and many similar single-point calculations exclusively capture the effect of the selected electronic structure treatment on the specific EPR observable while neglecting both thermal averaging and environmental effects on that very property. However, finite temperatures certainly activate ro-vibrational modes even under computational vacuum conditions, which in turn affect the electronic structure of the EPR probe molecule and, thus, all its properties including EPR parameters. The classical statistical averages of HFCCs of probe molecules in the gas phase at finite temperature conditions has some theoretical relevance, for example, as a useful approximation^26^ for vibrational corrections to calculations of HFCCs of quasi-rigid EPR probes with a negligible Boltzmann population of excited vibrational states.^27,28^

The next layer of complexity comes from the presence of the environment since EPR probes are mostly used to interrogate condensed matter systems, be they proteins, liquids, or solids at specific thermodynamic conditions. Arguably, the simplest such environment is a solution, aqueous environments certainly covering a vast range of real-life applications of EPR experiments. This poses the challenge to faithfully represent the impact of solvation, in particular, of hydrogen bonding in the specific case of aqueous environments, on the thermal average of *A*^iso^. The existing work of solvation effects on EPR observables has been repeatedly summarized in review articles.^13,29,30^ Over the years, different theoretical approaches have been developed to calculate EPR observables in solvating environments.^31−42^ These studies include solvent effects implicitly in the framework of continuum solvation models or by adding explicit solvent molecules (“microsolvation” approach) either on equal footing with the spin probe at the level of electronic structure or via a force field (hybrid, mixed quantum mechanical/molecular mechanical, QM/MM) approximation, or a mixture of both dubbed semi-continuum modeling.

From a theoretical point of view, a quite satisfactory approximation to EPR properties obtained from measurements in the liquid state certainly are fully atomistic molecular dynamics simulations based on the interactions obtained from accurate electronic structure calculations, from which the properties were also to be computed on equal footing in an ideal world. Typically, however, the crucial tasks of (i) generating the statistical ensemble (mostly using periodic boundary conditions within computer simulation) and of (ii) computing the EPR properties (mostly using finite systems within quantum chemistry) are decoupled, which offers the opportunity to use different methods for both tasks. Apart from that fundamental decoupling, a key caveat to theoretically treat solvation in this most explicit way is the accuracy at which the electronic structure problem can be solved in practice in the realm of full ab initio, QM/MM or force field molecular dynamics (FFMD) or Monte Carlo simulations. Early work along these lines^33^ was devoted to compute the EPR properties of the benzosemiquinone radical anion in liquid water at ambient conditions where on the order of 100 snapshot configurations have been extracted from Car–Parrinello simulations of that solution. In another study,^34^ such ab initio molecular dynamics (AIMD) simulations^43^ were performed for an H_2_NO molecule in liquid water using about 100 water molecules and 10 ps-long AIMD trajectories. The same study also showed that frozen density embedding can reliably model the solvent effects on EPR observables as gauged against AIMD simulations. Computation of the *A*^iso^ parameter of the nitrogen atom of nitroxide-based EPR probes in their solution environment has also been performed^35,36^ using a QM/MM approach where only the spin probe itself was treated at the electronic structure (QM) level while the solvation environment was represented using fully parameterized molecular mechanics (MM). Combining a microsolvation treatment of the spin probe with a polarizable continuum model (PCM) for longer distance solvation by embedding the spin probe together with a few solvent molecules, called semi-continuum modeling in some literature, has also been used in the context of calculating EPR observables with solvation effects.^37−39^ Such an integrated “QM/PCM” approach has been used in several studies where Car–Parrinello simulations of the probe in explicit solvents were performed to sample solvation configurations.^37−39^ Using a similar philosophy, polarizable force field simulations were employed to generate the ensemble of configuration snapshots for subsequent single-point EPR calculations.^40^

We close our appraisal of representative previous work by stressing that, so far, the EPR observables of the ensemble of snapshot configurations have been computed within density functional theory (rather than using open-shell correlated wavefunction-based methods) and that the AIMD simulations of the spin probe in bulk water to sample the required configurations have been performed using generalized gradient approximation (GGA) density functionals (rather than using hybrid functional spin-polarized AIMD). In a nutshell, theoretical methods with a superior description of both electronic and solvation structure are required to push forward the frontier of computing the EPR properties of spin probes in liquid-state environments and beyond.

In the current work, we advance the cutting edge by calculating the thermal average of the *A*^iso^ parameter of a solvated spin probe in bulk water using state-of-the-art methodologies from AIMD and, as a novel approach, liquid-state integral equation theory (EC-RISM, see below) to describe solvation combined with accurate quantum chemistry when it comes to computing the EPR parameters, also in comparison with an established continuum solvation model (CPCM), as outlined in more detail in the next section. In the sense of a converged proof-of-concept investigation, our target is to calculate this experimentally accessible observable for a representative EPR probe in an aqueous solution at ambient thermodynamic conditions as accurately as possible. We have chosen a nitroxide-based EPR probe, namely, HMI (2,2,3,4,5,5-hexamethylimidazolidin-1-oxyl, C_9_H_19_N_2_O), as depicted in Figure 2. This is one of the pH-sensitive EPR probes whose protonation state and, hence, its magnetic response depends on the pH of the medium. Experimentally, this type of spin label can be effectively used to investigate surface potentials, local polarity, or p*K*_a_ values, and, moreover, it could also be used successfully for in vivo studies.^44−46^

The remainder of this paper is organized as follows: Section 2 discusses the general methodological aspects pertaining to solvation and EPR calculations, followed by the theoretical background in Section 3 that leads us from the gas phase to a realistic, dynamic model of the solvated radical. We develop the electronic structure and spectroscopy of HMI in water in a step-by-step approach leading from coupled cluster level gas-phase electronic structure calculations to AIMD. Experimental and computational details are compiled in Section 4. The results and their discussion follow in Section 5 before we summarize and discuss our specific and general findings and error sources including a perspective on future developments in Section 6.

## 2 Overview

**Figure 1:**
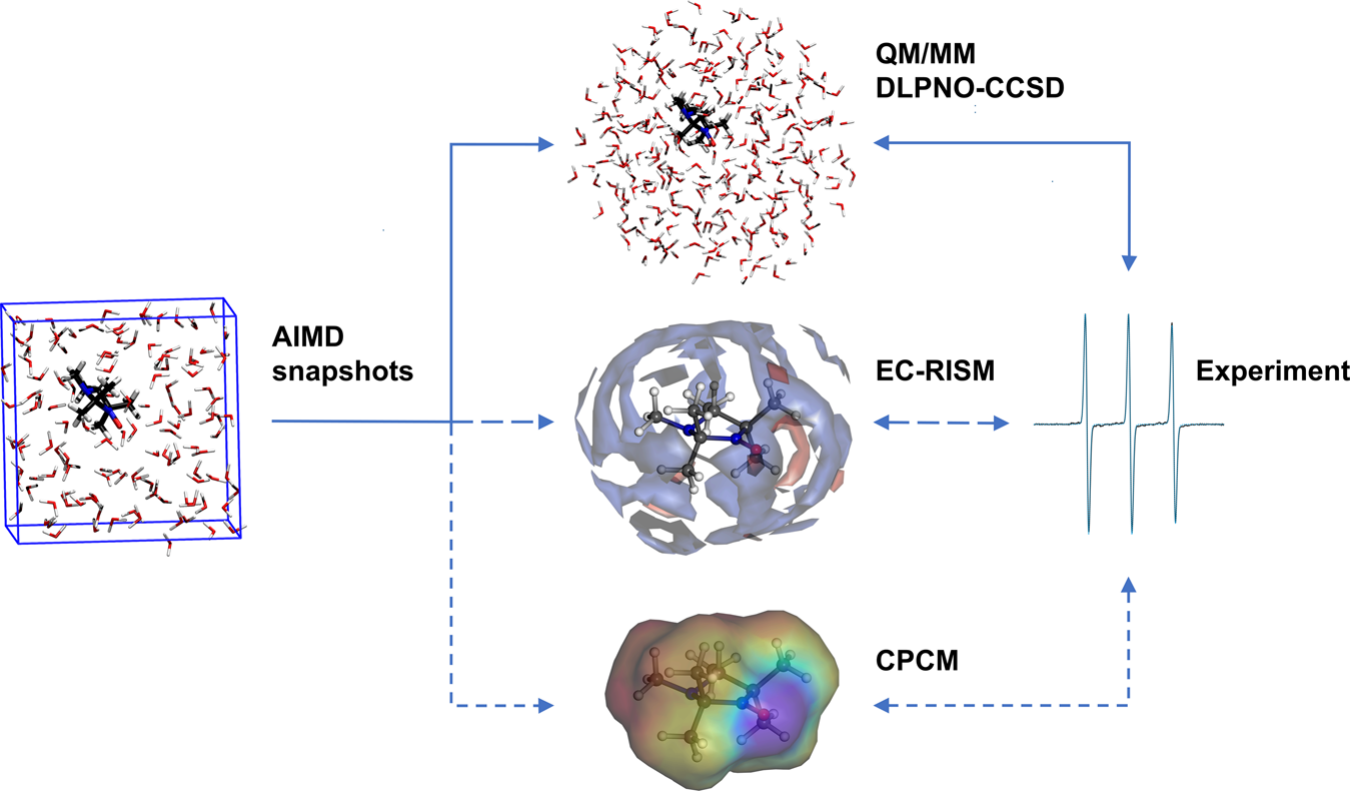
Outline of this study. The combination of AIMD and with accurately calibrated DLPNO-CCSD results leads to theoretical reference values of EPR properties that are compared to the experiment. The ability of the more efficient EC-RISM and CPCM approaches to reproduce the reference results will be critically assessed.

Since the present work reports a dedicated collaborative effort from researchers of different scientific communities that is of significant technical complexity, we will provide a brief overview of our strategy before entering into the details of the investigation. The general approach is schematically sketched in Figure 1.

In particular, we have performed substantial AIMD simulations of HMI in water using a spin-polarized hybrid density functional (revPBE0-D3) to generate the best possible ensemble of solvation configurations. The isotropic HFCC of the nitrogen (^14^N) and oxygen (^17^O) sites of HMI in aqueous solution was then obtained from single-point calculations using the open-shell variant of the domain-based pair natural orbital coupled cluster singles and doubles method (DLPNO-CCSD).^47−53^ For this purpose, solvation configurations have been extracted from the AIMD trajectory in a first step, and a QM/MM approach has been adopted to treat the most relevant molecules with DLPNO-CCSD theory (meaning HMI itself and increasingly large solvation shells are treated at the coupled cluster level), whereas all other solvent molecules have been included in terms of classical point charges at the sampled positions according to the AIMD configuration. In this way, we were able to carefully investigate into the convergence of the methodology (a) to the canonical CCSD limit and (b) to the basis set limit. Subsequently, we explored whether it is possible to obtain equivalent results by reducing the computational effort even further. To this end, the solvation environment was alternatively described in a statistical fashion at properly controlled thermodynamic conditions within the embedded cluster reference interaction site model integral equation theory (EC-RISM).^54−58^ Here, the equilibrium solvent structure (described by 3D RISM-approximated solute–solvent pair distribution functions for an MM water model) polarizes the electronic structure of the spin probe, which has the conceptual advantage of incorporating the intrinsic structure of the solvent. For this purpose, we extracted an ensemble of instantaneous snapshot configurations of HMI in water according to the AIMD trajectory but with all water molecules stripped off. We call such a structure of HMI “vertically desolvated” since it fully retains the structural memory of the local solvation environment in the bulk solution. The effect of retaining the statistical nature in modeling solvation is also explored by comparing the EC-RISM results with those of the hybrid density functional theory calculations using the standard continuum solvation embedding (CPCM)^59−61^ that only incorporates electrostatic effects through its dielectric constant. Very importantly, the ultimate accuracy gauge for our computations at this level of theory clearly is the experiment. We have therefore also performed X-band continuous wave (CW) EPR measurements to obtain accurate isotropic HFCC (*A*^iso^) toward the ^14^N of neutral (i.e., unprotonated) HMI in ultrapure water at very well-controlled thermodynamic and pH conditions.

**Figure 2:**
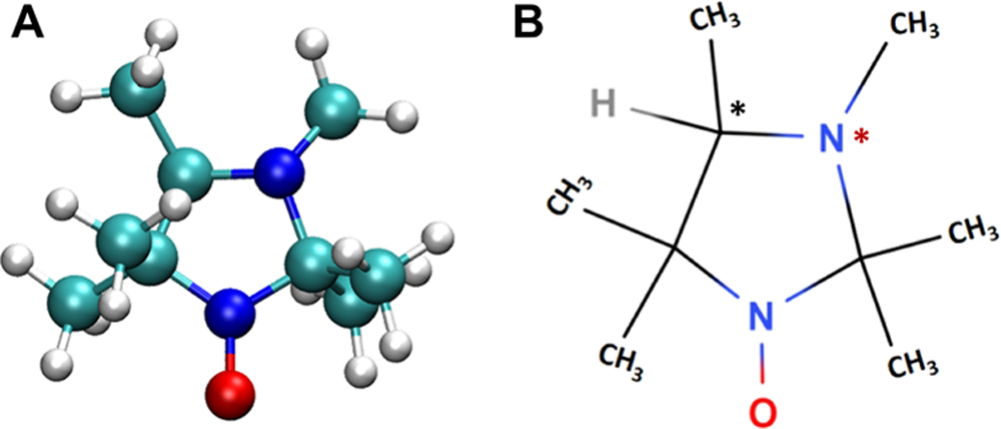
Three- (A) and two-dimensional (B) view of (3*R*,4*S*)-2,2,3,4,5,5-hexamethylimidazolidin-1-oxyl (HMI). The chiral carbon is marked with a black asterisk and the invertible chiral nitrogen atom with a red asterisk.

Our model system, HMI (see Figure 2), contains a chiral carbon center and an invertible chiral nitrogen, giving rise to two different diastereomers that are possibly found in a dynamic equilibrium. However, fixing the chiral carbon in the R configuration, only the (*R*)C–(*S*)N isomer is significantly populated (free energy difference in aqueous solution >3.6 kcal mol^–1^) due to the steric hindrance of attached methyl groups, which can be quantified by EC-RISM and CPCM calculations (see the Supporting Information, Table S1, along with the results from geometry optimizations in Tables S2–S4). Hence, only this dominant stereoisomer, which prevails during simulations, has been taken into account for high-level quantum chemical (QC) calculations.

## 3 Theory

### 3.1 Domain-Based Pair Natural Orbital Coupled Cluster Theory for Open-Shell Species (DLPNO-CCSD)

The canonical coupled cluster singles and doubles methods with perturbative triples method, CCSD(T), which has been dubbed the gold standard of quantum chemistry, very steeply scale as O(*N*^7^) with system size. The limited applicability of this method due to the computational expense has led to recent developments in local correlation approaches, which made coupled cluster methods affordable for large systems. These approaches exploit the fact that the correlation energy is the sum of pair energies which decrease rapidly with distance.^63−66^ Therefore, transforming the orbital description into a local basis reduces the costs drastically, especially when care is taken in the construction of the virtual space. The domain-based local pair natural orbital coupled cluster method, DLPNO-CCSD, uses pair natural orbitals (PNOs) to describe the virtual space.^47−53^ Making use of the sparse map data structure results in linear scaling and hence makes CC feasible for calculating accurate energies of large systems, which consist up to several hundred atoms.^67−70^ The derivation of the “Λ-equations” for closed- and open-shell DLPNO-CCSD furthermore enables the calculation of first-order response properties such as the HFCC.^71,72^

Here, we give only a brief overview on the calculation of the DLPNO-CCSD spin density as needed for HFCCs. For a detailed description, interested readers can refer to refs (67)–^72^. The CCSD energy functional is
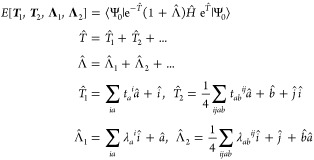
1with Ψ_0_ being usually the Hartree–Fock (HF) determinant, *Ĥ* is the Born–Oppenheimer Hamiltonian, and  are the standard fermion creation and annihilation operators for orbitals *q* and *p*, respectively. *T̂*(***t***) and Λ̂(**λ**) contain the coupled cluster excitation and de-excitation operators, respectively, with ***t*** and **λ** collectively denoting the cluster amplitudes and the Lagrange multipliers, respectively.

The first derivative of the energy functional can then be written as
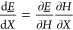
2provided that the functional is made stationary with respect to all parameters
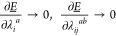
3
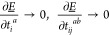
4where the latter set of equations are the “Λ-equations”. Furthermore, from the CCSD energy functional, one obtains the expression
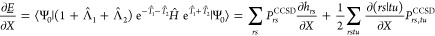
5where summation indices (*r*, *s*, *t*, *u*) refer to the orbital indices.  and  denote the one- and two-electron (written in Mulliken notation) integral derivatives, respectively. The perturbation-independent unrelaxed CCSD densities are determined by
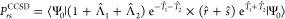
6
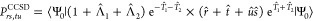
7

For the open-shell case, an α- and a β-density is obtained with their difference resulting in the spin density.

### 3.2 Calculation of Hyperfine Couplings

The spin Hamiltonian (in angular frequency units) for the interaction of an electron spin vector operator (**Ŝ**) with a magnetic nucleus N of spin (**Î**^(N)^) with an external magnetic field (**B**) is given by^73,74^
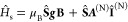
8where the first term represents the interaction between the electron spin and the external magnetic field. The constant μ_B_ is the Bohr magneton and the tensor ***g*** = *g*_e_**1**_3_ + Δ***g***, where Δ***g*** represents the correction to the free electron value *g*_e_ due to coupling of the orbital Zeeman and spin–orbit coupling operator, relativistic mass, and the gauge first-order corrections. The second term in the spin Hamiltonian describes the coupling between the total electron spin **Ŝ** and the nuclear spin of the nitrogen nucleus **Î**^(N)^ through the hyperfine coupling tensor ***A***^(N)^. For light nuclei, where the spin–orbit coupling can be ignored, the hyperfine coupling tensor has two parts
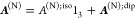
9where the first (scalar) term *A*^(N);iso^ is the isotropic Fermi contact interaction or HFCC. The second term, that is, ***A***^(N);dip^, describes the dipolar contribution to the HFCC, which is traceless and therefore not observable for rapidly tumbling species in fluid solution. It is well known that *A*^(N);iso^ is related to the spin density (ρ_N_) at the particular nucleus (Fermi contact term) and is given as
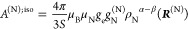
10where μ_N_ is the nuclear magneton and *g*_N_^(N)^ is the nuclear *g*-values of the nitrogen nucleus in question and ***R***^(N)^ is its position. *S* is the total spin of the state under investigation (*S* = 1/2 throughout this paper). The spin density at a given point ***r***, ρ_N_^α–β^(***r***) can be obtained from
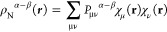
11where *P*_μν_^α–β^ is the difference between the spin-up (α) and spin-down (β) density matrices calculated at any given level of theory. In the rest of this paper, we drop the superscript N for the isotropic Fermi contact term and represent it as *A*^iso^.

As mentioned above and apparent from eq 10, the *A*^iso^ parameter depends on the spin density at the nucleus of interest. Accurate calculations of spin density require a very accurate wavefunction in the vicinity of the targeted nucleus. From the computational standpoint, the employed basis set should be flexible at the core region and capable of describing core level spin polarization accurately.^75^ The description of core-level spin polarization is catastrophic^76,77^ with Hartree–Fock theory. Low-order perturbation theory (e.g., second-order many body perturbation theory, MP2) cannot rectify these major shortcomings, and highly correlated wavefunction approaches, such as coupled cluster (CC) theory with single and double excitations (CCSD), in their traditional forms are computationally too demanding to be applied to most real-life problems. Thus, density functional theory based studies have been done with functionals from different rungs of Jacob’s ladder.^10−13,22−25^

There exists vast literature on calculations of the *A*^iso^ parameter for many EPR-active molecules using a broad array of different electronic structure methods in the realm of both density- and wavefunction-based theories. Static DFT-based calculations using the gas-phase equilibrium structure of EPR probes have been performed most extensively from the level of generalized gradient approximated (GGA), meta-GGA, and hybrid up to double-hybrid functionals.^10−13,22−25^ The ultimate conclusion from these studies turns out to be that hybrid functionals are best candidates for calculations of EPR properties of organic probes. Double-hybrid functionals do not improve the results for organic EPR probes over the hybrid class, but their application becomes more meaningful when there is a transition metal center present.^12^ As expected, canonical coupled cluster methods are found to be always superior to DFT for organic as well as metal-containing EPR probes.^12,14−21^ Given the overall goal of the present study, namely, reaching high accuracy for liquid-state systems, we opted to employ the open-shell coupled cluster approach in the framework of local pair natural orbital coupled cluster (DLPNO-CCSD) methods as detailed below.

### 3.3 Ab Initio Molecular Dynamics

The first step of an accurate calculation of thermal averages of EPR parameters for solvated spin probes, such as HMI in water, is to describe the solvation of such open-shell molecules on equal footing with the solvent molecules most accurately in a computationally feasible way. This will allow one to sample many realistic solvation configurations of finite size, the statistical ensemble of which can be treated using appropriate QC methods. In the framework of DFT-based AIMD, which is even today the only practical approach to extensively sample aqueous solutions, figuring out a functional that provides both reliable solvation properties and reasonably accurate EPR properties of open-shell species in water is an essential step.

When it comes to describing the properties of liquid water and aqueous solutions, there is a long tradition of using the computationally efficient functionals of the GGA family, such as BLYP or PBE to name but two, which overall provide a reasonable description of the properties of water. Clearly, they are unable to properly cope with open-shell solutes due to their self-interaction error (SIE), which generally produces artificial spin delocalization. In the case of aqueous solutions, this can lead to spin polarization within the solvation environment, which heralds unphysical and unacceptable artifacts in the description of the solvation shell properties of spin probes.^78^ However, it has also been demonstrated that this fundamental failure can be readily alleviated by correcting for the SIE.^78^ Among a wealth of distinct options to reduce or even eliminate the SIE, including the computationally economic Hubbard-*U* correction approach, hybrid functionals have been demonstrated to provide reasonable descriptions of spin densities and EPR parameters of metal-free open-shell molecules^12,13,22−25^ by replacing (semi)local exchange suffering from SIE by mixing in the rigorously SIE-free Fock exchange.

In particular, those functionals that belong to the class that has been designed to satisfy as many physical limits and bounds as possible, rather than being heavily parameterized, have been shown to provide reliable electron densities.^79,80^ Indeed, the well-established and thus well-tested PBE0 functional^81^ has been proven to provide overall a faithful description of properties of open-shell organic molecules including EPR parameters in the first place.^12,22−25^ Fortunately, a variant of that very functional, namely, revPBE0-D3 (see refs (81−83) for its three ingredients), has been demonstrated recently to yield an excellent description of the many-body potential energy surface of water which underlies the structural, dynamical, and spectroscopic properties of liquid bulk water at ambient thermodynamic conditions.^84^

Based on all these considerations and previous experience from very different fields of application, we consider revPBE0-D3 to be at this moment in time an excellent choice to reliably simulate the solvation properties of open-shell species, such as spin probe molecules, in bulk aqueous environments at ambient temperature and pressure conditions using practical AIMD simulations. We benchmarked spin-related properties of HMI, namely, its spin density and *A*^iso^ parameter using the equilibrium structure, against corresponding DLPNO-CCSD calculations (see Section 5). The favorable results that we obtain provide strong evidence that revPBE0-D3 is indeed an excellent dispersion-corrected hybrid functional to describe the solvation properties of spin probe molecules in aqueous environments.

### 3.4 Embedded Cluster Liquid-State Integral Equation Theory

Conducting AIMD simulations of periodic systems that host a solute species together with sufficiently many solvent molecules, say on the order of 100 H_2_O’s, based on hybrid functionals, such as revPBE0-D3, are computationally demanding. Long simulation times must be accessed to allow for exhaustive statistical sampling of solvation configurations to converge thermal averages of properties, such as the *A*^iso^ parameter sought here. A promising bridge between describing the solvated solute explicitly and on equal footing at the level of the electronic structure of the solution in the framework of AIMD on the one hand and continuum solvation approaches where the solvent is no longer described on a molecular basis on the other hand are solute embedding techniques that describe the presence of the solvent by molecular liquid-state integral equation theory, as reviewed in refs (85) and (86). In a nutshell, the molecularly explicit solvent environment around the solute is modeled at the level of two-point structural correlation functions of the inhomogeneous solution (akin to radial distribution functions). Thus, instead of describing solvation by explicit sampling of the thermal fluctuations of all explicit solvent molecules around the solute by running AIMD simulations, solvation in the framework of integral equation theory is described at the level of solute–solvent pair distribution functions of the inhomogeneous solvent with respect to the solute impurity, which encode the underlying thermodynamics. These distribution functions can be broken down to various levels of granularity implying different computational demands and approximation characteristics. Here, 3D RISM theory^87,88^ represents a well-balanced approach, where the solvent is represented by the set of its atomic, that is, site distribution functions on a 3D grid around the molecular solute. The resulting solvent charge distribution, taken from an established MM solvent model, can be allowed to polarize the solute’s electronic structure in a self-consistent manner, that is, the solute Hamiltonian is modulated corresponding to the given solvent distribution which in turn changes solute–solvent interactions. By iterating to self-consistency between electronic and solvation structure, we gain access to the full range of QC observables including HFCCs in solution.

Here, we only provide a short summary of the theory behind the EC-RISM approach to couple 3D RISM theory with electronic structure calculations, whereas more details can be taken from previous work.^54,55,87−89^ 3D RISM theory aims at calculating the well-known total correlation function between the solute and solvent sites interacting via the solute–solvent site potential *u*_γ_(**r**) at grid point **r** by solving a nonlinear system of equations, which consists of the integral equations
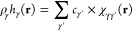
12and the closure relation
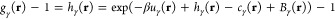
13where ρ_γ_ is the bulk phase density, *c*_γ_(**r**) is the direct correlation function, χ_γγ′_ is the precomputed pure solvent site–site susceptibility (density–density correlation function), β is the reciprocal temperature, and the asterisk denotes a convolution operation. *B*_γ_(**r**) is the bridge function which is generally unknown. The solute–solvent site pair distribution function *g*_γ_(**r**) is directly obtained from the total correlation function. The most common approximation is to neglect it, which leads to the “hypernetted chain closure” (HNC).^90,91^ Expanding the HNC closure by a *k*th-order power series in terms of the difference between *h* and *c* renormalized by the interaction potential leads to the PSE-*k* (partial series expansions) closure,^92^ which shows improvement in speed and numerical stability. The PSE-1 closure is also known as the “Kovalenko–Hirata” closure, while infinite order corresponds to the HNC approximation.^93^ In this work, the PSE-3 closure was exclusively used.

The term *u*_γ_(**r**) is described by the sum of Lennard-Jones (LJ) dispersion–repulsion interaction, where the corresponding parameters are taken from common force fields, and electrostatic interactions between solvent point charges, taken from the solvent force field, and the solute’s electrostatic potential (ESP) derived from the solvent-polarized wave function. A less computationally demanding alternative would be to use atom-based point charges fitted to the ESP, which has turned out to be less accurate in practical applications. These ESP-derived charges are only used as auxiliary quantities during iterations.^55^ The polarization effect of the solvent on the solute’s wave function is accounted for by discretizing the solvent charge density via
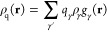
14to produce a set of embedding discrete background charges
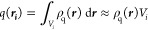
15whose interactions with nuclei and electrons can be represented by an electrostatic interaction operator *V̂* to be added to the unperturbed molecular electronic Hamiltonian *Ĥ*_o_
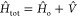
16

The polarized wave function changes the solute–solvent interactions which in turn yield modulated solvent distribution functions and corresponding embedding background charges. This cycle is iteratively repeated until a specific convergence criterion is met. Through this self-consistent mutual polarization of solvent structure and solute wave function, besides providing access to the solvation free energy, the effect of solvent on spectroscopic parameters can be accurately modeled, which was demonstrated in previous works ranging from ambient to extreme solvent conditions.^56−58^

### 3.5 Continuum Solvation Models

While EC-RISM is not yet routinely available as a solvation model in common QC software packages, continuum or “implicit” solvation approximations are readily provided. The physical basis of these models is the description of the solvent background as a structureless continuum represented by its dielectric constant, thus requiring the definition of a boundary (“cavity”) separating the molecule under investigation with its “internal” dielectric permittivity from the environment. Several variants have been conceived and implemented to couple the electronic structure problem to the polarization exerted by the dielectric continuum, most commonly in the form of PCM. We refer the reader to highly comprehensive reviews^94,95^ for further details and can focus here on the relation to EC-RISM calculations. Briefly, and in agreement with the EC-RISM approach, the electronic wave function is polarized by an external ESP attributed to the solvent, which, in the continuum solvation case, is calculated by solving the Poisson equation relating the charge density and the potential, subjected to boundary conditions at the cavity surface. Iterating to self-consistency between electronic structure and the polarization potential gives rise to an effective, purely electrostatic, solvation free energy along with the possibility to calculate spectroscopic properties from the polarized wave function.

For the specific case of the conductor-like PCM (CPCM)^59−61^ used exclusively in this work, the technically demanding boundary conditions are simplified by first replacing the “real” finite solvent dielectric constant by infinity, representing an electric conductor, and scaling the surface potential back to the required medium value, as originally introduced in the form of the “conductor-like screening model” (COSMO).^62^ Analogously to eq 16, the electronic Hamiltonian is perturbed by an ESP that is mapped under conductor-like boundary conditions via
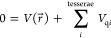
17to polarization charges *V*_q*i*_ that are related to the solute’s ESP *V*(*r⃗*), located on the so-called tesserae embedding the molecule’s cavity. The vector of the conductor-like polarization charges **Q** is determined via
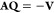
18with **V** containing the ESP of the solute acting on the tesserae whose specific characteristics are provided as matrix **A**.

From an implementation point of view, PCM and EC-RISM calculations are therefore related, as in the former case, the effect of the surface potential that completely encodes the electrostatic embedding from solving Poisson’s equation is mapped onto discrete point charges located on the tesserae that “solvate” the Fock matrix, similar to the embedding of the electronic wave function in the field of background charges representing the solvent distribution from RISM calculations. The “solvation” Fock matrix element can in either case be written as
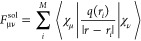
19with *M* representing the number of tesserae (PCM) or regularly spaced background charges (EC-RISM). While RISM makes use of LJ potentials to avoid penetration of solvent charges into the core regions, the PCM cavity is constructed from empirically adjusted atomic radii.

## 4 Experimental and Computational Details

### 4.1 Experimental Details

The HMI spin probe was synthesized from 2,2,4,5,5-pentamethylimidazolin-1-oxyl^96^ as previously described.^97^ Briefly, dimethylsulfate (2.4 mL, 25.8 mmol) was added to a solution of 2,2,4,5,5-pentamethyl-3-imidazolin-1-oxyl (2.0 g, 12.9 mmol) in dry diethyl ether (40 mL). The solvent was removed under vacuum, and the residue was left under vacuum (on a rotary evaporator) with a bath temperature of ca. 40 °C for crystallization (0.5–1 h). The solid residue was triturated with dry diethyl ether, and the crystalline precipitate was filtered off and washed with dry diethyl ether. The obtained crude 2,2,3,4,5,5-hexamethylimidazolinium-1-oxyl methylsulfate salt (3.26 g, 11.6 mmol, 90%) was dissolved in 40 mL of ethanol, and sodium borohydride (0.66 g, 17.4 mmol) was added to the stirred solution portionwise. The reaction mixture was stirred for 40 min, then ethanol was evaporated under vacuum, and the residue was dissolved with 20 mL of water and extracted with chloroform (3 × 20 mL). The combined extract was dried with sodium carbonate and evaporated to dryness under vacuum. The residue (1.88 g) was purified by sublimation under vacuum to yield 1.8 g (91%) of HMI as an orange crystalline solid with a melting point of 40–42 °C.

**Figure 3:**
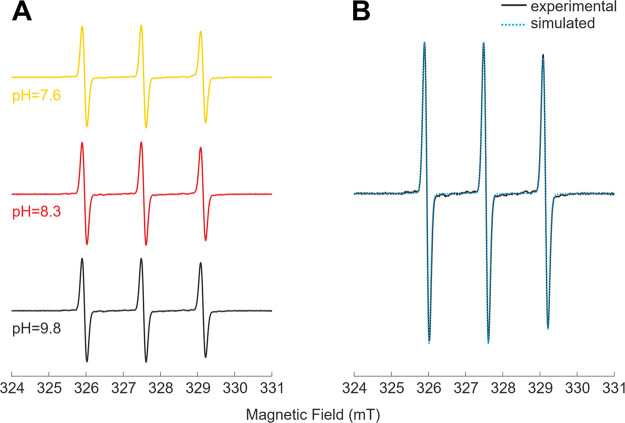
(A) X-Band CW EPR spectra recorded at 295 K of unprotonated HMI in aqueous solutions at three different pH values (pH ≫ p*K*_a_) detected with the same spectrometer. The three spectra are identical. (B) Comparison between the experimental (black, pH 9.8) and EasySpin-simulated (dotted blue) spectra, see the text for details.

For the EPR experiments, a first solution containing 100 μM HMI (from a stock solution of 5 mM) and 100 μM NaOH (from a stock solution of 1 M) was prepared in ultrapure Milli-Q water. The pH was controlled with a pH-meter (FiveEasy Plus FP20 from Mettler Toledo) and found to be 9.8. We further diluted this solution by adding the corresponding volume from a previously prepared HMI 100 μM solution, yielding other two samples with final pH values of 8.3 and 7.6. The spin concentration of HMI in the solutions was verified by comparing the double integral of the EPR spectrum of HMI with that obtained on a reference TEMPOL solution at a known concentration. After measuring the pH, the samples were immediately transferred into the EPR tubes, which were sealed with wax to avoid changes in pH due to additional CO_2_ dissolving slowly from the air. X-Band CW EPR measurements were carried out on a Bruker ELEXSYS E580 X-band spectrometer equipped with a Bruker ER 4122HSQ super-high *Q* cavity. The temperature was kept constant at 295 K with a Bruker liquid nitrogen variable temperature unit. Additionally, the spectrum at pH 10 were also recorded in a second Bruker ELEXSYS E500 X-band spectrometer equipped with a Bruker ER 4122HSQ super-high *Q* cavity. The samples were loaded in capillaries of 0.9 mm inner diameter, and the spectra were acquired with the following parameters: 9.2 GHz microwave frequency, 0.47 mW power, 100 G sweep width, 0.8 G modulation amplitude, 117.19 ms conversion time, 1024 points, and 1 scan. All EPR spectra were simulated using the MATLAB routine EasySpin^98^ and the subroutine garlic with the following parameters: *A*^iso^ = 44.87 MHz, line width = 0.12 mT, and τ_corr_ = 25 ps, see Figure 3.

### 4.2 DLPNO-CCSD Calculations

All single-point property calculations were conducted using the ORCA quantum chemistry package, version 4.2.^99−101^ HFCCs for isotopes ^14^N and ^17^O were calculated using revPBE0-D3, B3LYP-D3,^102^ and DLPNO-CCSD electronic structure in conjunction with the def2-TZVPP basis set^103^ with decontracted s-functions. Note that quantum mechanically calculated EPR spectroscopic observables are independent from the D3 dispersion correction added to the density functional since it does not affect the electronic structure; however, it was employed to be consistent with the AIMD simulations where including the D3 correction is known to generally improve the description of water and aqueous solutions. Tight convergence thresholds, no frozen core approximation, and the RIJK approximation for the two-electron integrals were applied. For the DLPNO-CCSD calculations, the correlation auxiliary basis set was chosen to be cc-PWCVTZ/C,^104^ and the parameters for a special treatment of the core region in the DLPNO scheme was set according to the “Default2” settings in ref (74). These settings are based on the detailed study of generating accurate spin densities for first-order property calculations such as HFCCs at the DLPNO-CCSD level. For the QM/MM approach, the solvation complexes as given by the AIMD snapshots (see Figure 6) were separated into a subsystem containing the whole nitroxy spin label and all water molecules up to the second solvation shell (thus defining the QM subsystem which is treated with DLPNO-CCSD theory) and the remaining water molecules (thus defining the MM subsystem) which describe the electrostatic field in which the QM subsystem is embedded. The electrostatic field due to the solvent water molecules in the MM region was obtained by using the point charges of the TIP3P water model^105^ placed at the positions of the water molecules in the respective AIMD configuration. The computer timings provided in the Results section are based on running a development version of ORCA 5.0 on 8 Intel Xeon E5-2690v2 3.0 GHz cores with a 6 GB RAM per core.

### 4.3 AIMD Simulations

We have performed AIMD simulations of one neutral HMI molecule in a cubic periodic box of 128 water molecules using Born–Oppenheimer propagation.^43^ The initial configuration of the solutions as well as the box length was obtained from preliminary FFMD simulations. The TIP4P/2005 model^106^ was employed for water, whereas the force field for HMI (Table S6) and computational details on FFMD are deferred to Supporting Information Section S2.

These AIMD simulations were performed using the CP2K code^107,108^ wherein the electronic structure was solved using its QUICKSTEP^109^ module. The atom-centered TZV2P Gaussian basis set in conjunction with Goedecker–Teter–Hutter pseudopotentials^110−112^ and a plane wave basis with a kinetic energy cutoff of 500 Ry were used to represent the Kohn–Sham orbitals and total electron density, respectively. Acceleration to compute the Fock exchange terms of the revPBE0-D3 hybrid functional was achieved by applying the auxiliary density matrix method^113^ with the cpFIT3 auxiliary basis. We note in passing that we have cross-checked our AIMD simulation parameters by evaluating the bulk water structure (128 water molecules in an approximately 15.67 Å cubic supercell such that the water density is close to 1.0 g/cm^3^ at 300 K) using the revPBE0-D3 functional, resulting in favorable agreement with the recent literature.^84^ The D3 dispersion correction^83^ was applied, both in CP2K and ORCA, using zero damping and considering only the two-body terms.

For the simulations of the open-shell solute in water, we have solved the spin-polarized Kohn–Sham equations for HMI in water. The simulations were performed in the *NVT* ensemble using massive Nose–Hoover chain thermostatting^114^ where each and every Cartesian coordinate gets is own thermostat. The equations of motions were integrated using a timestep of 0.5 fs. The total length of the AIMD trajectory was 206 ps, of which the first 6 ps was the equilibration phase subsequent to pre-equilibration by FFMD. From the rest of the 200 ps of the AIMD trajectory, solvation configurations were extracted after every 200 and 500 fs for calculating *A*^iso^ using DFT and DLPNO-CCSD methods, respectively. Thus, the reported thermal averages are based on DFT and DLPNO-CCSD calculations of the *A*^iso^ parameters using 1000 and 400 solvation configurations, respectively. We refer the reader to Section 5.2.3 for our protocol to extract these snapshots.

### 4.4 EC-RISM Calculations

The EC-RISM calculations were performed on a cubic grid with 120^3^ points and a grid spacing of 0.5 Å. The water solvent susceptibility was taken from ref (89) (modified SPC/E model) using a dielectric constant of 78.4 and a number density of 0.0333295 Å^–3^.^56^ The dielectric constant of bulk water used within CPCM and EC-RISM are slightly different, which is even quantitatively irrelevant for all practical purposes. The LJ parameters were taken from GAFF force field version 1.4 and are listed in the Supporting Information (Table S7).^115^ The convergence criteria were set to 10^–6^ for the maximum residual norm of direct correlation functions within 3D RISM calculations and to 0.01 kcal mol^–1^ for the maximum excess chemical potential difference between two consecutive EC-RISM cycles. All EC-RISM calculations were performed with ORCA 4.2.1 to solve the electronic structure of the embedded cluster. In all iterations, the revPBE0-D3 approach in conjunction with the def2-TZVPP basis set^103^ with decontracted s-functions was used. Exact periodicity-corrected electrostatics extracted from the electron density were utilized for the DFT calculations.^54,56^ The auxiliary atom-centered point charges were calculated with the CHelpG scheme using Breneman–Wiberg radii^116^ with a 0.3 Å grid spacing and a maximum distance of all atoms to any grid point of 2.8 Å, an additional restraint to reproduce the QC-derived dipole moment was not applied. Clusters of point charges representing the solvent were merged together using a hierarchical algorithm inspired by the Barnes–Hut treecode method.^117^ The essence of this algorithm is that the number of charges that are collapsed into a single-point charge grows with increasing distance to the nearest solute atom. This greatly reduces the number of embedding point charges. Only in the last iteration, after convergence of the EC-RISM cycle, the EPR property calculation was performed. These DFT-based EC-RISM and CPCM calculations were applied to both the microsolvated clusters taken from AIMD simulations and the corresponding vertically desolvated HMI structures in order to delineate the performance of implicit and RISM-based solvation models to account for the solvation effect on EPR parameters in comparison with explicitly solvated conformations.

Additionally, as a new development, our EC-RISM code was combined with the DLPNO-CCSD method within ORCA. At variance with the DFT-based EPR calculations within EC-RISM solvation, where solvation complexes of the solute with nearby water molecules attached have been embedded, only the vertically desolvated HMI structures were used for these much more demanding DLPNO-CCSD calculations. During the iterations, HF calculations were performed to represent the electronic structure of HMI and electrostatic solute–solvent interactions; only after convergence of the EC-RISM cycle, the DLPNO-CCSD calculation was utilized to add electron correlation effects in the liquid-state environment for the calculation of spectroscopic properties. The other settings within EC-RISM remained untouched.

In addition, the difference between CPCM and EC-RISM solvation models was evaluated for the minimum free energy structure of HMI exposed to the respective solvent models. Using similar protocols as in previous studies,^55,57,58^ geometry optimizations of HMI with CPCM solvation using the revPBE0-D3 functional were first performed (Table S4). Using the optimized structures, *A*^iso^ parameters of the nitrogen and oxygen atoms of the nitroxy group of HMI under CPCM and EC-RISM solvation with revPBE0-D3 and DLPNO-CCSD were calculated. The purpose of this optimization is to determine the influence of using the single minimized equilibrium structure of HMI under continuum solvation on the EPR observables versus using the ensemble of HMI snapshots from AIMD which all deviate from its equilibrium structure due to thermal fluctuations. In previous publications,^55,57,58^ usually only optimized minimum free energy structures were considered to quantify the impact of solvation, so a comparison between these two approaches seems to be useful at this point at a small additional cost. The aforementioned parameters and technical settings were used for calculating the *A*^iso^ parameters. Recall that all nonperiodic electronic structure calculations within this work have been carried out consistently using the def2-TZVPP basis set.^103^

### 4.5 CPCM Calculations

We have also performed implicit solvent EPR property calculations using CPCM^59−61^ to treat solvation with the revPBE0-D3 electronic structure. Within that model, the solvation properties of water are reduced to and thus captured by its bulk dielectric constant of 80.4, which is the default dielectric constant used in ORCA version 4.2.1. The solvent-excluding surface was constructed using the GEPOL algorithm with a default probe radius of 1.3 Å and a minimal distance between two adjacent surface points of 0.1 Å. To investigate the robustness of the CPCM calculations, we varied the probe radius within revPBE0-D3/def2TZVPP/CPCM//revPBE0-D3/def2TZVPP/CPCM calculations, yielding *A*^iso^ values of 30.0530, 30.0240, 30.0143, and 30.0000 MHz for radii of 1.1, 1.2, 1.3, 1.4 Å, respectively, thus demonstrating the negligible impact on the results reported in this study.^1^

## 5 Results and Analysis

### 5.1 Experimental Results

Since our core aim is to critically scrutinize a cutting edge computational approach to compute the average HFCCs of a specific spin probe in water at ambient condition, thus taking solvation and temperature effects properly into account, against the experimental value of the *A*^iso^ parameter for the nitrogen of HMI in water, we first discuss the experimental results. Note that the oxygen of the nitroxide contributes negligibly to the signal due to the small abundance of oxygen isotopes with nonzero nuclear spin, though we later also report calculated numbers of the respective HFCC, which could be a useful reference when ^17^O-enriched samples are used. The p*K*_a_ of HMI was previously found^118^ to be around 4.5 at ambient temperature. A comparison of the different CW X-band CW-EPR spectra at different pH values >7 is shown in Figure 3. At these conditions, the spectra exhibit an identical shape with only one spectral component present, proving that the nitroxide moiety is not protonated, indicating that HMI is in its neutral charge state as assumed in the computations. Note that a residual presence of protonated species would lead to the appearance of another spectral component with a smaller *A*^iso^, which is mostly visible as a shoulder at the high-field pure water solutions.^118^

The experimental spectra obtained at pH ∼ 10 with two different spectrometers were simulated with EasySpin, and the error in the *A*^iso^ value has been estimated to be equal to half of the spectral resolution (0.14 MHz). In Table 1, the *A*^iso^ parameters obtained in this work are compared with reference literature data and are found to be in close agreement. Due to the consistency of our measurements, we decided to define the experimental benchmark value of the *A*^iso^ parameter of nitroxide N in unprotonated HMI in liquid bulk water at ambient conditions (295 K) to be 44.87 ± 0.14 MHz.

**Table 1:** Comparison of *A*^iso^ Values of Nitrogen Atom of HMI in Water Obtained in This Work and as Found in Earlier Experiments by X-Band CW-EPR at Room Temperature

*A*^iso^ (MHz)	references
45.08 ± 0.06	(119)
45.16 ± 0.14	(120)
45.08 ± 0.14	(121)
44.87 ± 0.14	this work
44.87 ± 0.14	this work

### 5.2 Electronic Structure Calibration

#### 5.2.1 Computational Convergence of Coupled Cluster-Based Hyperfine Couplings

We performed extensive benchmarking of different computational parameters with the aim of evaluating their impact on the DLPNO-CCSD calculations. The HMI molecule under vacuum was optimized based on revPBE0-D3/def2-TZVPP (Table S5), and that optimized structure has been used for the following tests. The evaluation of the technical setup was separated into three steps, focusing on one parameter at a time: (1) basis set, (2) auxiliary basis set, and (3) property settings. The data on which optimization of these settings is based is compiled in Tables 2, 3, and 4, respectively.

**Table 2:** Basis Set Convergence Study for DLPNO-CCSD Calculations of the *A*^iso^ Parameter of Optimized HMI under Vacuum with Fixed Auxiliary Basis Set, Full Electron Treatment, and Different Contraction Schemes of the Basis Functions Contracted = Contraction of the Whole Basis Set, Decontracted-s = Decontracted s-Functions of the Basis Set, Decontracted-b = Decontraction of the Whole Basis Set

basis set	contraction scheme	time	*A*^iso^(N) (MHz)
def2-SVP	contracted	29 min	65.4
	decontracted-s	56 min	25.5
	decontracted-b	63 min	24.7
def2-TZVPP	contracted	4.8 h	27.1
	decontracted-s	7.1 h	29.6
	decontracted-b	12.4 h	29.5
def2-QZVPP	contracted	1.9 days	29.6
	decontracted-s	2.7 days	29.1
	decontracted-b	3.6 days	29.7
cc-PCVDZ	contracted	48 min	24.1
	decontracted-s	78 min	30.0
	decontracted-b	99 min	28.7
cc-PCVTZ	contracted	12.4 h	28.5
	decontracted-s	16.1 h	29.3
	decontracted-b	15.8 h	29.5
cc-PCVQZ	contracted	4.1 days	30.1
	decontracted-s	4.7 days	29.8
	decontracted-b	5.1 days	29.5

**Table 3:** Auxiliary Basis Set Study of the *A*^iso^ Parameter of Optimized HMI under Vacuum Using def2-TZVPP, Full Electron Treatment, Decontracted-s, and DLPNO-HFC1^a^

auxbasis	time (d:h:m:s)	*A*^iso^(N) (MHz)
autoaux	9.7 h	29.6
cc-PWCVTZ/c	7.1 h	29.6
def2-TZVPP/c	7.1 h	29.4

a The DLPNO-HFC1 settings correspond to the “Default1” DLPNO-CCSD settings for accurate spin densities of ref (71).

**Table 4:** Property Setting Study of the *A*^iso^ Parameter of Optimized HMI under Vacuum Using def2-TZVPP, Full Electron Treatment, Dectontracted-s, cc-PWCVTZ/c with DLPNO-HFC1 Corresponding to the “Default1” Setting, and DLPNO-HFC2 to the “Default2” Setting of Ref (71)

property setting	time	*A*^iso^(N) (MHz)
DLPNO-HFC1	7.1 h	29.6
DLPNO-HFC2	15.6 h	30.1

Our benchmark results are summarized in Table 2. A distinct difference is observable between the double-ζ and triple-ζ basis sets, whereas the change from triple-ζ to quadruple-ζ drops to only 1 MHz or less, which lies within the fundamental error of the method as such. Hence, one can consider the values obtained with the quadruple-ζ basis set to be very close to the complete basis set limit. Furthermore, HFCCs are very sensitive to the description of the core region, which renders a full electron treatment crucial for the calculation of this property. Additionally, a distinct difference of the HFCCs can be observed between the contracted and decontracted-s scheme for the def2 basis sets, whereas the difference between decontracted-s and decontracted-b is fairly small. Note that the difference between the contracted and decontracted schemes is not as pronounced for the cc-PCVXZ basis sets since they already describe the core region more rigorously by including core polarization functions. This is also the reason for the small improvement when going from cc-PCVDZ to cc-PCVTZ compared to the analogous change of the def2-basis sets.

To summarize, the def2-TZVPP basis set with decontracted s-functions in combination with the cc-PWCVTZ/c auxiliary basis set and an all electron treatment and property settings according to the “Default2” setting of ref (71) gives the best results for the HFCC computation using DLPNO-CCSD in the sense of balancing computational cost versus accuracy most efficiently. It reproduces the most accurate results obtained with a very large, decontracted basis set including core polarization functions (decontracted cc-PCVQZ) with a remarkable accuracy of 0.1 MHz while leading to turnaround times that are about 20 times faster. This technical setting is used for all subsequent calculations that are reported in the remainder of this paper.

#### 5.2.2 Comparison of revPBE0-D3 DFT Results with DLPNO-CCSD: Hyperfine Couplings and Spin Density Distribution

Although we have employed the revPBE0-D3 functional in the AIMD simulation due to its ability to reproduce the solvation structure of bulk water, it is necessary to have an estimate of its ability to give spin properties of HMI. Hence, we have first considered how well the revPBE0-D3 functional performs with respect to the DLPNO-CCSD method as far as the spin properties of HMI in the gas-phase equilibrium structure (being the optimized structure mentioned in Section 5.2.1) are concerned. Since the computational settings in CP2K and ORCA are different, we compared the spin properties with CP2K/revPBE0-D3, ORCA/revPBE0-D3, and ORCA/DLPNO-CCSD combinations. The revPBE0-D3 functional was applied using the specific AIMD settings of CP2K as well as using the single-point QC settings of ORCA as reported in Sections 4.2 and 4.3, respectively, for calculations of spin properties. Note that nonperiodic CP2K calculations were performed specifically for this purpose using the wavelet Poisson solver^122,123^ as the DLPNO-CCSD calculations performed with ORCA are also of nonperiodic nature.

**Figure 4:**
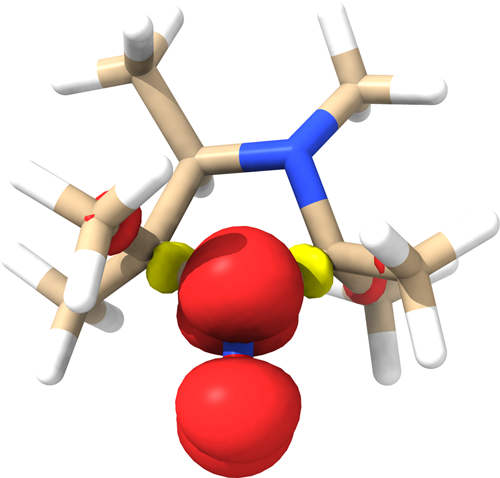
Spin densities on the nitrogen and oxygen atoms of the NO moiety of the optimized isolated HMI molecule from DLPNO-CCSD calculations (cutoff ±0.003 electrons/*a*_0_^3^, red: positive, yellow: negative). Images are optically indistinguishable from revPBE0-D3 results computed by ORCA or CP2K, see the text.

Specifically, we have compared the spin density on the nitrogen and oxygen atoms of the nitroxy moiety. The spin densities of the isolated HMI molecule calculated using the DLPNO-CCSD electronic structure are depicted in Figure 4. The spin density plots with revPBE0-D3 was indistinguishable with naked eyes. The Mulliken spin populations at the oxygen and nitrogen atoms (N/O) according to revPBE0-D3 using ORCA and CP2K are approximately 0.44/0.51 and 0.51/0.49, respectively, while they are 0.40/0.60 according to DLPNO-CCSD. Finally, the *A*^iso^ parameter of nitrogen is calculated to be 27.5 and 30.1 MHz with the revPBE0-D3 and DLPNO-CCSD methods (both computed with ORCA), respectively. The corresponding *A*^iso^ parameters of the oxygen atom are −41.3 and −55.8 MHz, respectively. Thus, the revPBE0-D3 functional yields qualitatively satisfactory spin densities and EPR properties of HMI under vacuum, which is expected to also hold true in aqueous solution.

#### 5.2.3 AIMD: Solvation Behavior of HMI in Water and Extraction of Solvation Configurations

**Figure 5:**
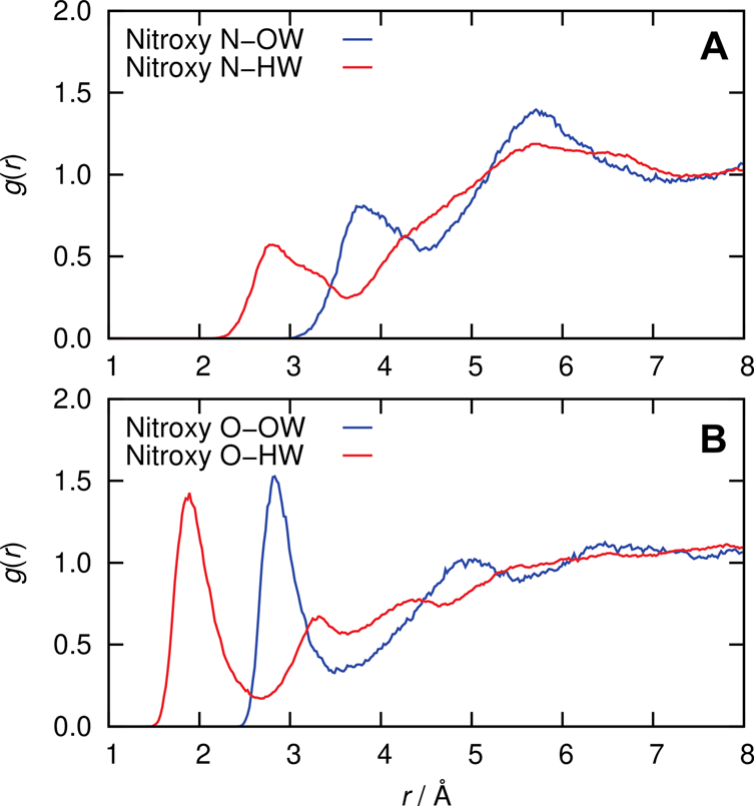
Radial distribution function of the water oxygen (OW) and hydrogen (HW) sites with respect to the (A) nitroxy oxygen and (B) nitroxy nitrogen of HMI in an aqueous bulk solution from AIMD simulations at ambient conditions.

The average solvation behavior around the nitroxide moiety of HMI in water, NO, which is the site of the unpaired electron, is analyzed in terms of the radial distribution functions shown in Figure 5. The radial distribution of water oxygen with respect to the oxygen of HMI has a distinct minimum at about 3.4 Å, which defines the first solvation shell. There is some weak structuring around the HMI oxygen ranging from 3.4 to 5.5 Å, which heralds the second solvation shell.

**Figure 6:**
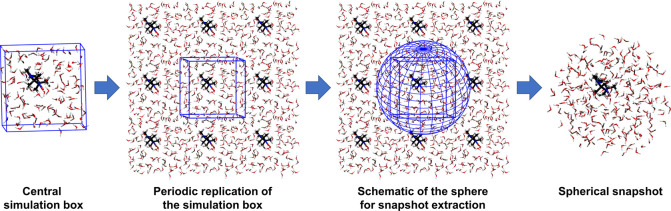
Scheme applied for extracting snapshots from the AIMD trajectory, see the text. Note that the oxygen site of HMI is considered as the center of the snapshots.

In order to calculate EPR parameters from electronic structure based on explicit solvation of HMI in water, we have extracted snapshots from the AIMD trajectory after every 200 fs such that these configurations are somewhat uncorrelated and, thus, provide a useful statistical ensemble of independent snapshots. There is a caveat to the process of extracting snapshots from periodic cubic supercells (as in CP2K) and subsequently performing electronic structure calculations using nonperiodic finite clusters (as in ORCA). We came up with the following simple protocol as illustrated schematically in Figure 6. First, the parent supercell has been periodically replicated in all three spatial dimensions, where the oxygen site of HMI defines the center of the coordinate system. Note that the oxygen of HMI is the heavily hydrated site with the unpaired electron. Second, we have considered a sphere which circumscribes all water molecules outside the first solvation shell of the oxygen site of all replicated HMI molecules in all neighboring periodic images as shown in the third panel of Figure 6. This allows us to extract a fairly large cluster containing about 275 water molecules where the most solvated site of HMI, namely, the oxygen of the nitroxide moiety, is at the respective center; note that this avoids clashes with the first solvation shells of any HMI replica but includes periodically replicated water positions and thus correlations with respect to the solute species. This procedure is applied to all snapshot configurations that have been extracted from AIMD to calculate the *A*^iso^ value in the realm of explicit solvation in conjunction with QM/MM (Section 5.2.4) embedding of the explicitly treated solvation water molecules around HMI.

#### 5.2.4 Convergence of Coupled Cluster Hyperfine Calculations Using Explicit Solvation Snapshots

In order to calculate the EPR parameters of HMI in water for very many configuration snapshots that contain a large number of explicit water molecules, it is simply impossible to treat the whole system consisting of the spin probe and all solvating water molecules in the AIMD simulation cell using open-shell DLPNO-CCSD calculations. Thus, we have decided to take recourse to a QM/MM embedding method where only the most relevant water molecules along with HMI are to be treated including full electronic structure (QM), while the vast majority of the solvent molecules is represented by a set of point charges (MM) at the proper positions as given by the respective snapshot (using the partial charges according to the TIP3P water model). However, to decide a criterion for choosing water molecules to be included in the QM subsystem, some reference system is needed that allows us to benchmark the QM/MM approximation in the first place. Thus, two independent random snapshots of HMI in water were chosen from AIMD simulations as the reference systems which were treated using full DLPNO-CCSD. We emphasize that each of these reference systems contains 415 atoms in total, of which 31 belong to the spin probe and the rest belongs to water (i.e., 128 water molecules). We denote these two reference systems as “reference system I” and “reference system II”. Note that the reference systems were chosen directly from the AIMD trajectory without resorting to the spherical snapshot scheme of the previous section (Figure 6) and thus differ from the snapshots of the established workflow. As the sole purpose of choosing the two reference systems is to benchmark the local solvation configurations to be considered in the QM region, there is already a sufficient number of water molecules in each frame of the AIMD trajectory. Furthermore, instead of the nitroxy oxygen, the center of mass of HMI was chosen as the center of each of two frames (reference systems), which does not influence the convergence of the QM region, again due to the sufficient number of solvent molecules.

Applying the resulting technical setup as worked out in Section 5.2.1, calculations of the two *A*^iso^ parameters were conducted for both reference systems. Each calculation took 65 days on eight Intel(R) Xeon(R) CPU E5-2687W v4 @ 3.00 GHz cores with a 42 GB memory per core. The resulting HFCC of the nitroxy nitrogen (oxygen) for what we call the “HMI + full solvation” setup was 44.3 (−51.8) and 45.2 (−50.0) MHz for reference system I and reference system II, respectively. These reference values are to be compared to in the following when assessing the QM/MM approximation. We note that such close agreements between the chosen random snapshots and the experimental value is serendipitous. The fair deal is to compare the ensemble averaged value of *A*^iso^ with that of the experimental result using a well-controlled QM/MM treatment of the explicit solvent embedding as follows.

Having these reference systems at our hand, we varied next the number of water molecules that are included in the QM subsystem. In the asymptotic limit of including more and more water molecules in the QM subsystem, we anticipate that *A*^iso^ approaches the value obtained for the reference system. Thus, we extracted HMI together with a certain number of water molecules from the reference system in the sense of a systematically improvable approximation to the latter. One model included the water molecules of the first solvation shell (HMI + first solvation shell) and the other model included all water molecules up to the second solvation shell in the QM region (HMI + second solvation shell), while all the remaining water molecules were treated in the MM region as electrostatic point charges of the TIP3P water model. For the sake of demonstration, a QM region consisting solely of bare HMI itself was considered as well, keeping the full QM/MM embedding in the field of point charges (HMI no solvation shell). Some representative images of spherical snapshots are shown in the Supporting Information (Figure S3).

The computed HFCCs for the nitroxy nitrogen and oxygen sites of HMI are presented in Table 5 for three different QM/MM embedding approximations compared to the “full solvation” reference limit. Comparison of the *A*^iso^ values obtained when using the QM/MM model with only HMI in the QM region versus using the optimized equilibrium structure of HMI under vacuum (dubbed “gas phase”) makes clear that the purely electrostatic embedding of the spin probe in the solution already accounts for the majority of the solvation shift of the N site with respect to vacuum conditions. The effects of adding the explicit first and second solvation shell/s to the QM region are less pronounced, but nonetheless non-negligible at the desired accuracy scale. At the same time, the computational effort is reduced from 65 days for the all-QM DLPNP-CCSD calculation in the “full solvation” limit to roughly 1 day for the corresponding QM/MM approximation while not sacrificing any accuracy.

**Table 5:** Calculations of the *A*^iso^ Parameter of the Nitroxy N/O Sites of HMI in Water Using the Reference Snapshot Configurations I and II (See Text) Based on QM/MM DLPNO-CCSD Single-Point Calculations Using Different QM Regions (See Text) Regarding the Inclusion up to the *n*th Solvation Shell (* Calculated on 8 Cores and ** Calculated on 16 Cores, See Text)^a^

QM region	reference system	time (h)	*A*^iso^(^14^N/^17^O) (MHz)	Δ_ref_(^14^N/^17^O) (MHz)	#H_2_O treated with DLPNO-CCSD theory
HMI + full solvation	reference system I	1560	44.3/–51.8		128
	reference system II	1200	45.2/–50.0		128
HMI no solvation shell	reference system I	11*	42.4/–53.2	1.9/1.4	0
	reference system II	16*	44.8/–50.8	0.4/0.8	0
HMI + first solvation shell	reference system I	15**	44.1/–52.3	0.0/0.5	2
	reference system II	19*	44.8/–50.6	0.4/0.6	1
HMI + second solvation shell	reference system I	36**	44.3/–51.7	0.0/0.0	12
	reference system II	39**	45.1/–50.2	0.1/0.2	16

a Note that DLPNO-CCSD yields *A*^iso^ values of 30.1 and −55.8 MHz for the nitrogen and oxygen atoms, respectively, for the gas-phase equilibrium structure of HMI (optimized using revPBE0-D3/def2-TZVPP). Δ_ref_ = |*A*_HMI+full solvation_^iso^ – *A*_HMI+reduced solvation_^iso^| where either “no solvation shell” or “1st solvation shell” or “second solvation shell” as mentioned in the table refers to reduced solvation.

#### 5.2.5 Summary of Calibration Studies

To summarize, the most reliable QM/MM scheme contains the HMI molecule along with the water molecules up to the second solvation shell of the oxygen site of the nitroxy group included in the QM region, whereas the rest of the water molecules are treated as TIP3P point charges at the positions of the O and H sites of the water molecules in the respective snapshots. All subsequent calculations of the *A*^iso^ parameters were employing this QM/MM scheme in conjunction with the def2-TZVPP/decontracted-s basis set and different electronic structure methods as specified. The corresponding distribution functions of the *A*^iso^ parameters and their thermal averages in solution were subsequently calculated based on the full snapshot ensembles as specified earlier.

### 5.3 Thermally Averaged Hyperfine Couplings in Solution from AIMD: DLPNO-CCSD versus Hybrid DFT

**Figure 7:**
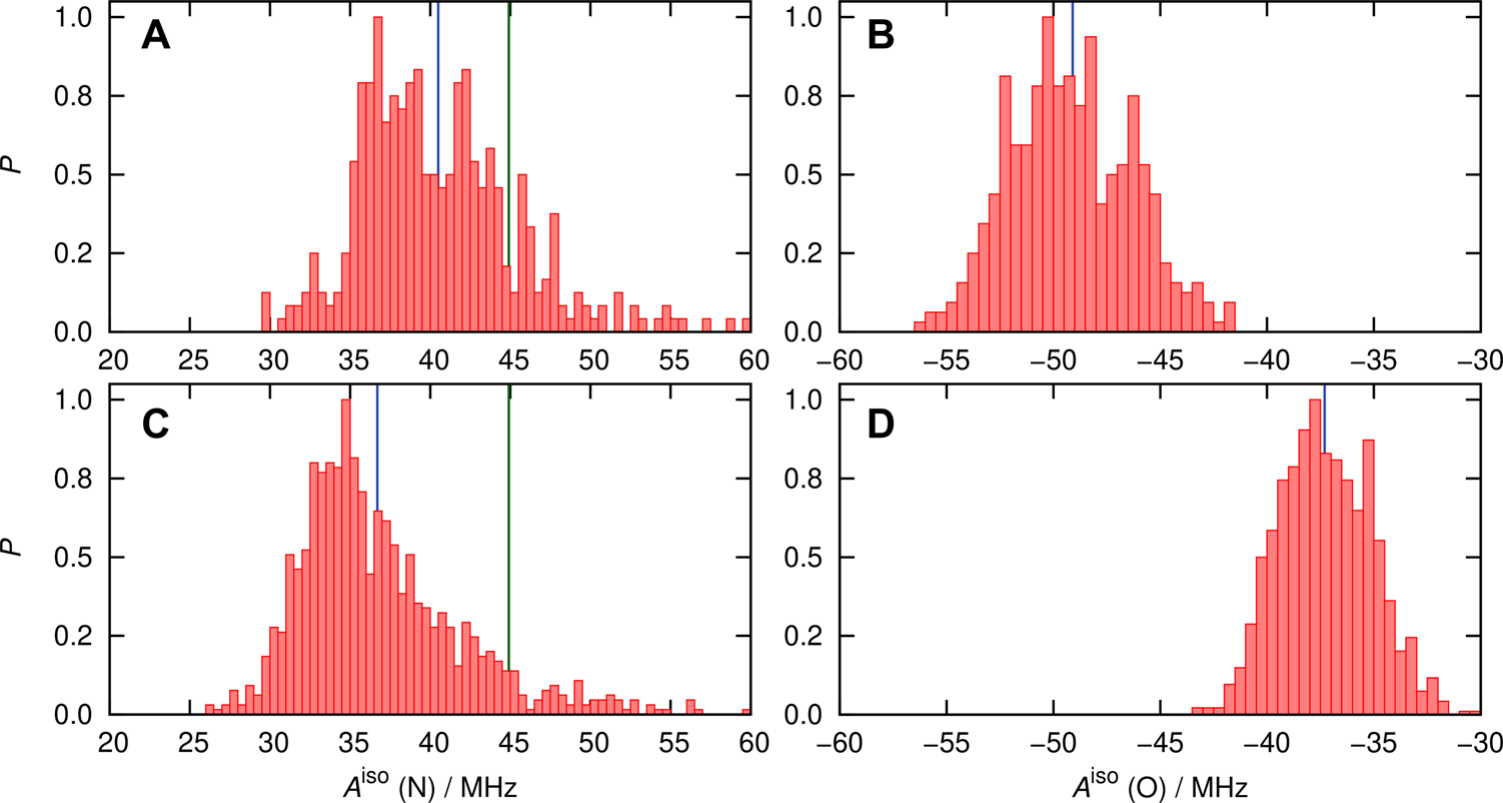
Probability distributions (normalized by setting the respective maximum bin values to 1) of the *A*^iso^ values of nitroxy nitrogen (left panels) and oxygen (right panels) of HMI in water at ambient conditions based on using the ensemble of HMI solvation complexes (spherical snapshots, Section 5.2.3) as sampled from AIMD simulations, see the text. Data in panels (A,B) are obtained from DLPNO-CCSD (averages 40.5 and −49.2 MHz) and (C/D) from revPBE0-D3 (averages 36.7 and −37.3 MHz), see the text. The blue line in each panel represents the average value of the corresponding distribution computed using the numerical *A*^iso^ data that underlie the respective histogram. The DLPNO-CCSD and DFT-based distributions are obtained from calculations over 400 and 1000 snapshots, respectively, as explained in the text. The green vertical lines in panels A and C represent the experimental benchmark value of 44.87 ± 0.14 MHz.

The *A*^iso^ values of the N (^14^N) and O (^17^O) atoms of the nitroxy group were calculated from DLPNO-CCSD theory (QM region: HMI + second solvation shell) together with classical point charge embedding (MM region: all solvent molecules beyond the second solvation shell) based on the QM/MM protocol of Section 5.2.4 using the spherical solvation configurations extracted from the AIMD trajectory of HMI in water by employing the approach outlined in Section 5.2.3. Note that the reference systems (I and II) of Section 5.2.4 were obtained directly from the AIMD trajectory as cubic snapshots and used only to benchmark the appropriate QM and MM regions. However, all subsequent calculations have been accomplished by employing the QM/MM protocol (benchmarked in Section 5.2.4 on the spherical snapshots obtained according to the protocol of Section 5.2.3). The probability distribution functions of this parameter are shown in Figure 7.

The distributions are quite broad, which can be attributed to the fluctuations in the structure of both the spin probe molecule and the solvation environment. The DLPNO-CCSD value of the *A*^iso^ parameter obtained by averaging over all underlying configurations (400 snapshots) is 40.5 MHz at 300 K while the corresponding experimental value is 44.87 ± 0.14 MHz. We have also compared the distributions obtained with hybrid functional calculations of this EPR parameter in Figure 7. The ensemble-averaged value (1000 snapshots) of the *A*^iso^ parameter with revPBE0-D3 is 36.7 MHz. Clearly, the coupled cluster approach provides a significant improvement over the tested hybrid functional with reference to the experimental benchmark value. The corresponding values for the oxygen atom of the nitroxy group are found to be −49.2 and −37.3 MHz, respectively, with DLPNO-CCSD, revPBE0-D3 treatments. We consider these thermal averages of the *A*^iso^ parameter of the spin probe in aqueous solution to be the best we can currently achieve upon combining rigorous statistical mechanics and correlated wavefunction-based electronic structure theory.

**Figure 8:**
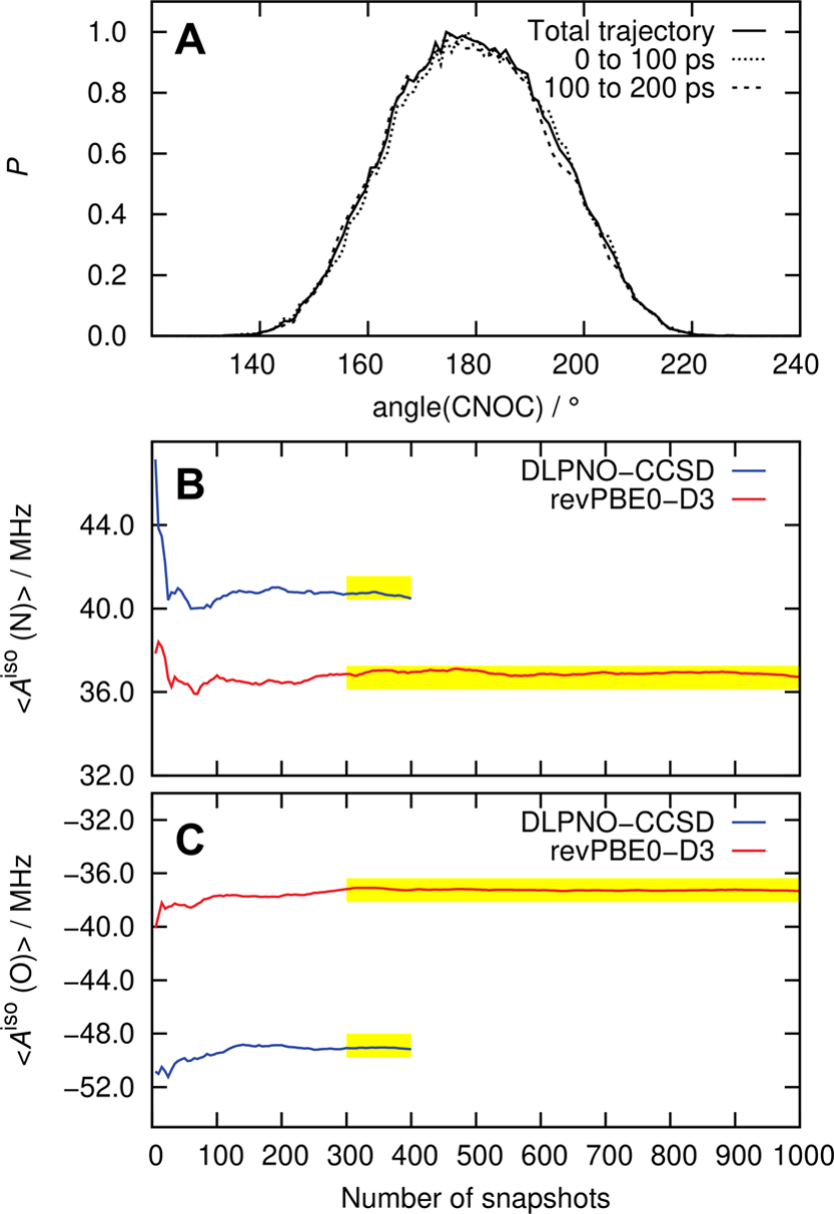
Convergence study of (A) thermal distribution function (normalized by setting the respective maximum bin values to 1) of the improper NO dihedral angle spanned by the CNOC group based on splitting the total trajectory into the first and second halves and of the average value of the *A*^iso^ parameter of HMI (B) nitrogen and (C) oxygen with respect to the sample size obtained from calculations with spherical snapshots treated according to the QM/MM protocol, see the text.

A prerequisite to the calculations of these thermal distribution functions and the resulting averaged values is the statistically sufficient sampling of the improper dihedral angle of the NO group (i.e., the CNOC angle) during the AIMD simulation, which is well known to greatly impact the thermal averages of *A*^iso^ values of spin probes in water as demonstrated recently using a tailor-made QM/MM sampling approach.^35^ We show in Figure 8A that the thermal distribution function of the CNOC angle is sufficiently converged for subsequent property analysis by splitting the entire AIMD trajectory (of 200 ps after equilibration used to compute the reported thermal averages) into its first and second halves.

We have also checked the convergence of the average value of the two *A*^iso^ parameters with respect to the number of solvation configurations or sample size in Figure 8B,C. The average values turned out to be essentially converged after about 300 snapshots. Upon comparing to the averages obtained by considering up to 1000 configurations in case of the less demanding DFT calculations, we estimate statistical sampling errors of approximately 0.5 and 0.1 MHz for the thermal average of *A*^iso^ for the nitrogen and oxygen sites of HMI, respectively, as visualized by the yellow bars in Figure 8B,C on the appropriate scale. Thus, we report the average *A*^iso^ values that have been obtained from 400 and 1000 snapshot calculations using DLPNO-CCSD and DFT electronic structure calculations, respectively.

### 5.4 Comparison of AIMD and EC-RISM Results

Although the DLPNO-CCSD calculations with explicit solvation water achieve considerable accuracy, this comes at a high price since many single-point calculations need to be carried out. Thus, we have assessed the performance of two solvation models, namely, EC-RISM and CPCM with respect to computing the thermal average of *A*^iso^ for HMI in bulk water. First, we want to start with a comparison between AIMD and EC-RISM.

**Figure 9:**
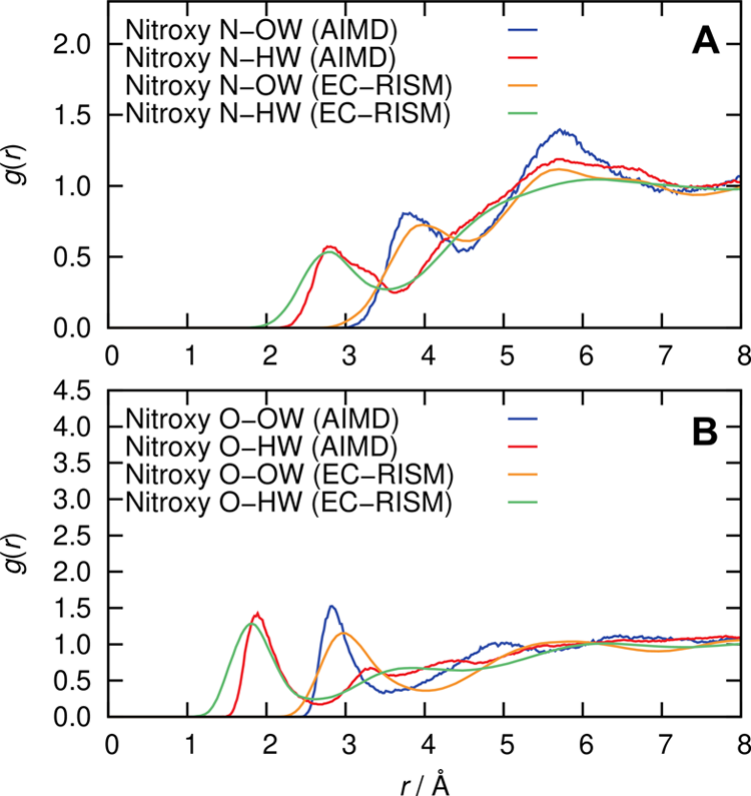
Radial distribution functions of water oxygen (OW) and hydrogen (HW) sites with respect to the (A) nitroxy nitrogen and (B) nitroxy oxygen of HMI in aqueous bulk solutions, calculated from AIMD simulations and from EC-RISM integral equation theory using revPBE0-D3/def2-TZVPP/EC-RISM on the optimized structure (CPCM, Table S4) at ambient conditions.

Therefore, we have first compared the radial distribution functions obtained from AIMD and EC-RISM solvation of HMI in water in Figure 9, which show close correspondence and emphasize the realistic representation of solvent structuring around the HMI obtained from EC-RISM calculations. Before applying our coupled cluster technique, the computationally much more efficient revPBE0-D3 functional has been used to this end, starting with geometry optimization of HMI within the CPCM solvation model. Note that these represent geometries in solution that differ from the optimized HMI structures in the gas phase considered in Sections 5.2.1 and 5.2.2. Using this optimized structure together with EC-RISM solvation, the *A*^iso^ parameter of the N atom was calculated using revPBE0-D3 and DLPNO-CCSD theory. The *A*^iso^ values for the nitrogen were found to be 32.5 and 38.1 MHz for revPBE0-D3 and DLPNO-CCSD (see Table 6), respectively. Remarkably, the difference between DLPNO-CCSD and revPBE0-D3 of 5.6 MHz agrees well with the corresponding difference from thermal averaging (Section 5.3) over the AIMD trajectory of 3.8 MHz. Hence, when we neglect the impact of HMI’s thermal fluctuations by comparing relative trends only, not absolute accuracy compared to experiment, EC-RISM appears to properly reflect the solvation effect on electronic structure.

**Table 6:** Comparison of Thermal Averages of *A*^iso^ of HMI in Water at Ambient Conditions Using Different Electronic Structure Methods to Compute This EPR Property in Conjunction with Using Different Solvation Approaches; the Values in the Absence of Any Thermal and Solvation Effects as Obtained for the Optimized Structure of HMI under Vacuum Are Reported for Comparison^a^

electronic structure method	solvation approach	*A*^iso^ of ^14^N/^17^O (MHz)	corresponding figures
DLPNO-CCSD	HMI at the gas-phase equilibrium structure	30.1/–55.8	
	HMI/explicit water up to the second solvation shell/rest of the solvent atoms of the spherical snapshots as MM-point charges	40.5/–49.2*	7A/B
	vertically desolvated HMI/EC-RISM	42.7/–47.8*	12A/B
	vertically desolvated HMI/CPCM		
	vertically desolvated HMI	33.4/–55.5	
	HMI at the solution equilibrium structure with EC-RISM solvation	38.1/–47.9	
revPBE0-D3	HMI at the gas-phase equilibrium structure	27.5/–41.3	
	HMI/explicit water up to the second solvation shell/rest of the solvent atoms of the spherical snapshots as MM-point charges	36.7/–37.3	7C/D
	vertically desolvated HMI/EC-RISM	37.0/–37.4	10C/S4C
	HMI/explicit water up to the second solvation shell/EC-RISM	37.5/–36.6	10D/S4D
	vertically desolvated HMI/CPCM	34.4/–39.2	11C/S4E
	HMI/explicit water up to the second solvation shell/CPCM	36.7/–37.3	11D/S4F
	vertically desolvated HMI	31.2/–41.2	10B/S4B
	HMI at the solution equilibrium structure with EC-RISM solvation	32.5/–37.6	
	HMI at the solution equilibrium structure with CPCM solvation	30.0/–39.4	

a The asterisk (*) marks our best computational estimate that serves as the intrinsic benchmark for more approximate calculations in comparison with the experimental benchmark value of 44.87 ± 0.14 MHz.

**Figure 10:**
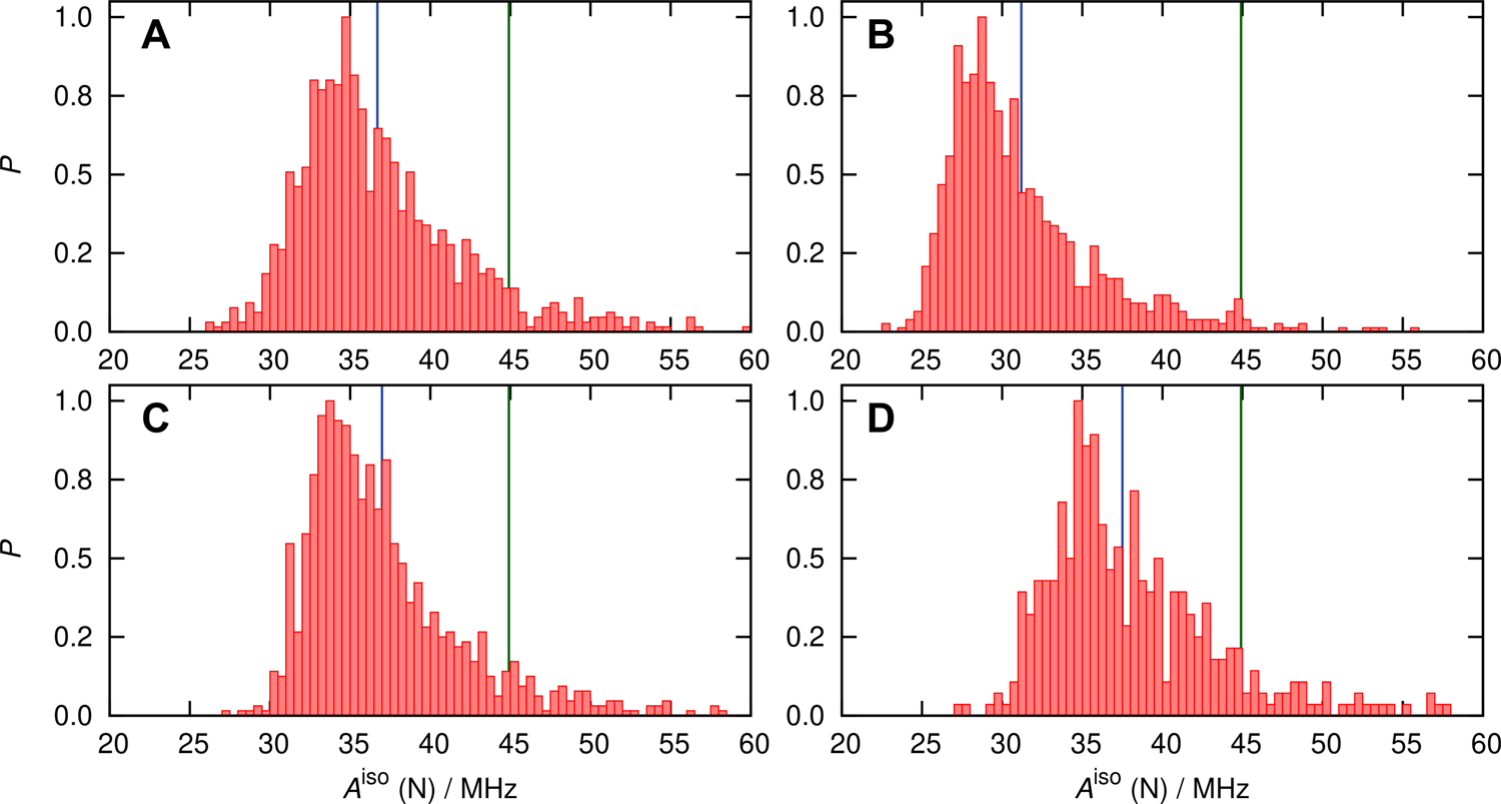
Probability distributions (normalized by setting the respective maximum bin values to 1) of the *A*^iso^ values (calculated with the revPBE0 D3 functional over 1000 snapshots) of the nitroxy nitrogen of HMI (A) with explicit water configurations [spherical snapshots, Section 5.2.3 treated according to the QM/MM (average 36.7 MHz) protocol] and (B) vertically desolvated HMI (average 31.2 MHz) configurations, (C) with EC-RISM solvation (average 37.0 MHz), and (D) with water molecules up to the second solvation shell around the HMI oxygen with EC-RISM solvation (average 37.5 MHz). The blue line in each panel represents the average value of the corresponding distribution computed using the numerical *A*^iso^ data that underlie the respective histogram. The green vertical lines represent the experimental benchmark value of 44.87 ± 0.14 MHz.

Next, we extracted the vertically desolvated configurations of HMI and also clusters of HMI with water molecules including up to the second solvation shell of the nitroxy oxygen using the same set of AIMD snapshot structures (1000 snapshots) as used in the previous section for the coupled cluster benchmark. The set of snapshots were then treated using the EC-RISM solvation model to evaluate the *A*^iso^ parameter of the nitrogen and oxygen atoms of the nitroxy group. In Figure 10, the distributions of the *A*^iso^ values of the N-atom for the vertically desolvated structures as well as for the ones containing the first two solvation shells are depicted for the revPBE0-D3 functional. Note that panel (A) reports the data obtained from explicit solvation (as already shown in Figure 7C) and, thus, serves here as the internal benchmark to describe solvation when using the revPBE0-D3 single-point electronic structure to compute the *A*^iso^ values (average 36.7 MHz). Similarly, panel (B) serves as our internal benchmark for vertically desolvated HMI under vacuum, thus neglecting solvation effects but still considering thermal effects, that is, intramolecular vibrational motion, on the molecular skeleton of HMI in solution at 300 K. Thus, the difference between these two averages, 36.7 and 31.2 MHz, of +5.5 MHz quantifies—in that sense—the solvent shift of the isotropic HFCC of the nitroxy nitrogen of HMI in water at 300 K. For the oxygen atom, the revPBE0-D3 functional (Supporting Information Figure S4) yields a shift of +3.9 MHz of *A*^iso^ from solvated to vertically desolvated states of the probe. Panel (C) shows the effect of EC-RISM solvation on the vertically desolvated structures, resulting in an average *A*^iso^ value of 37.0 MHz. EC-RISM solvation leads to a substantial shift compared to the vertically desolvated structures under vacuum with a difference of 5.8 MHz. Thus, the EC-RISM-derived water distribution is able to capture solvation effects on the isotropic HFCC, similar to the explicit model with two solvation shells and remaining water represented as point charges. Inclusion of the two first solvation shells around the N–O motif explicitly within EC-RISM calculations changes the *A*^iso^ value almost negligibly to 37.5 MHz, emphasizing that EC-RISM alone reproduces the largest part of solvation effects on HFCCs properly. However, thermal averaging is an essential ingredient for approaching quantitative accuracy as the predicted value for the EC-RISM-solvated single optimized HMI structure, 32.5 MHz (see also Table 6), deviates more strongly from the benchmark DFT-derived *A*^iso^ value of 36.7 MHz.

### 5.5 Comparison of AIMD and CPCM Results

Next, we address the effect of continuum solvation on *A*^iso^ compared to the AIMD and EC-RISM data. We start with the *A*^iso^ values of the nitroxy nitrogen calculated for the optimized geometry using the revPBE0-D3 functional. Remember that at the current stage, DLPNO-CCSD calculations in combination with CPCM are not available within ORCA 4.2.1. The *A*^iso^ value for the revPBE0-D3-optimized structure with CPCM solvation is 30.0 MHz (see Table 6) and thus 2.5 MHz smaller than the corresponding value obtained by EC-RISM. Note that CPCM describes the solvent at the level of a structureless continuous medium, whereas EC-RISM captures the structural heterogeneity of solvents, which appears to be essential for adequately modeling the solvation effect on HFCCs.

**Figure 11:**
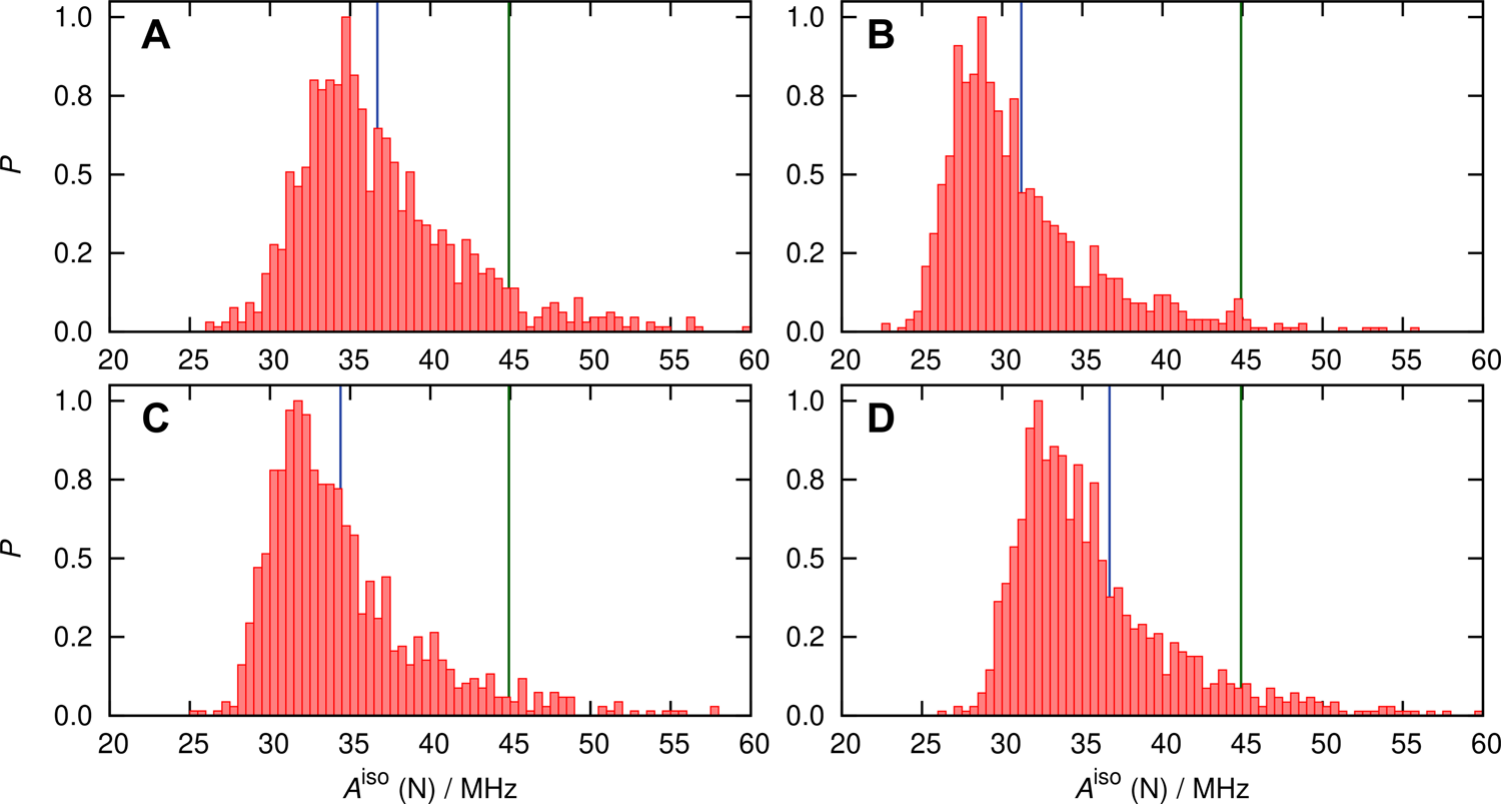
Probability distributions (normalized by setting the respective maximum bin values to 1) of the *A*^iso^ values (calculated with revPBE0-D3 functional over 1000 snapshots) of nitroxy nitrogen of HMI (A) with explicit water configurations [spherical snapshots, Section 5.2.3 treated according to the QM/MM protocol (average 36.7 MHz)] and (B) vertically desolvated HMI (average 31.2 MHz) configurations, (C) with CPCM solvation (34.4 MHz), and (D) with water molecules up to the second solvation shell of HMI (average 36.7 MHz) oxygen with CPCM solvation. The blue line in each panel represents the average value of the corresponding distribution computed using the numerical *A*^iso^ data that underlie the respective histogram. The green vertical lines represent the experimental benchmark value of 44.87 ± 0.14 MHz.

Figure 11 represents an analogue of Figure 10, with the difference that here the CPCM results are compared with, on the one side, the data obtained from explicit solvation with additional point charges (as already shown in Figure 7C) and on the other side vertically desolvated HMI under vacuum, where the solute–solvent interactions are completely neglected. For the vertically desolvated structures (1000 snapshots) (panel C), a mean value for the HFCCs of 34.4 MHz is obtained for CPCM solvation, resulting in a deviation of 2.3 MHz from the value obtained from explicit solvation with additional point charges (36.7 MHz). However, a significant improvement (+3.2 MHz) compared to the solely vertically desolvated structures (31.2 MHz) is observed. Once the first two solvation shells are explicitly included in the calculations, the result is significantly improved even with CPCM. In this manner, the CPCM solvation with explicit water molecules gives the same value as obtained from placing the explicit water molecules of the first two solvation shells around HMI and representing the remaining water molecules by point charges.

### 5.6 Comparison of EC-RISM and CPCM Results

Comparing vertically desolvated HMI after re-solvation by EC-RISM and the CPCM continuum approach unveils that the former fully captures the solvent shift of +5.5 MHz. By contrast, continuum solvation provides a shift of +3.2 MHz and hence captures only about 60% of the solvent effect. Continuum solvation can indeed quantitatively describe the solvent shift of *A*^iso^, but only after including the first and second solvation shells, whereas RISM embedding yields essentially the same shift as already obtained without considering any explicit solvent molecules. Thus, emphasizing the results from the previous subsection, EC-RISM solvation is powerful in the sense that the underlying solvent distribution from RISM integral equation theory captures very well the solvation of HMI in ambient water even without considering additional explicit water molecules in the electronic structure calculations in the effective field exhibited by the RISM-derived solvent distribution. This finding also agrees with the observation in Section 5.2.5 that the QM/MM DLPNO-CCSD model accounting for even the closest water molecules solely by their point charge effect on the solute’s electronic structure captures most of the solvation effect on the HFCCs. One can therefore conclude that charge-transfer effects between the solute and the solvent play a negligible role for this EPR parameter of HMI in water, at least in the neutral state treated in this work.

## 6 Overall Comparison of Calculated Solvation Effects on EPR Properties

In the preceding section, we have first compared carefully calibrated electronic structure calculations along with carefully calibrated AIMD trajectories with experiment and found satisfactory agreement. In the following, we have investigated the ability of the EC-RISM and CPCM models to reproduce these reference results. Our findings clearly show that EC-RISM embedding on the vertically desolvated structures can faithfully reproduce the effect of explicit solvent water of the two first solvation shells, whereas CPCM continuum embedding of the vertically desolvated structures misses the apparently important effects of structural heterogeneity of the solvent around the spin probe. The remaining difference of about 8 MHz with respect to to the experimental value can likely be explained by the choice of the electronic structure method used to compute the EPR parameter. To assess the impact of improving the underlying theory, DLPNO-CCSD as used previously in explicit solvation calculations was also combined with EC-RISM re-solvation of the ensemble of vertically desolvated HMI structures.

**Figure 12:**
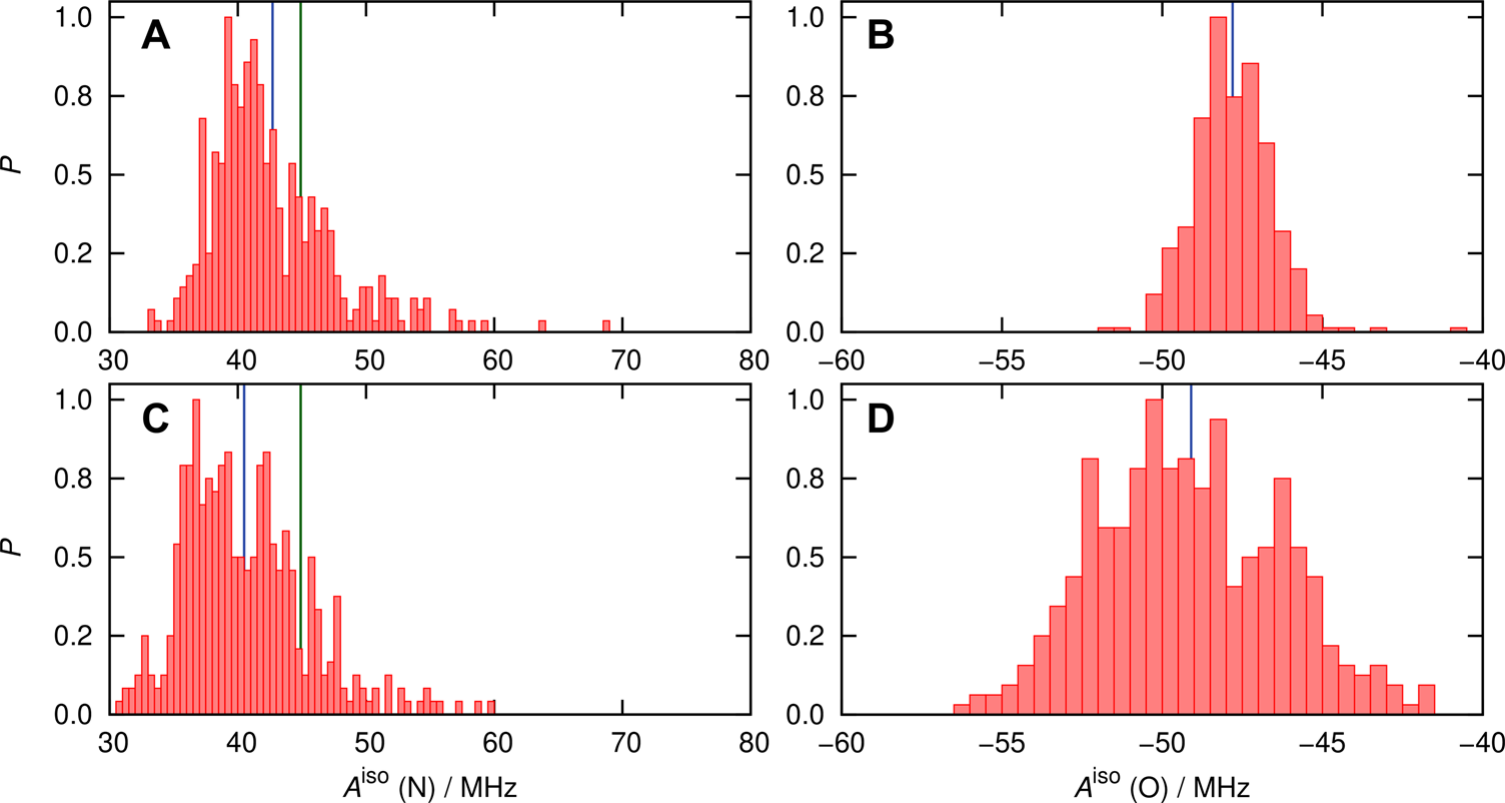
Probability distributions (normalized by setting the respective maximum bin values to 1) of the *A*^iso^ parameter of the (A) nitrogen (average 42.7 MHz) and (B) oxygen (average −47.8 MHz) of HMI calculated with DLPNO-CCSD theory using the AIMD ensemble of 400 vertically desolvated spin probe configurations with subsequent EC-RISM embedding for re-solvation of the spin probe. Panels (C,D) are the corresponding [to (A,B), respectively] distributions of *A*^iso^ values for HMI in explicit water according to the QM/MM protocol. Note that panels (C,D) are the same as Figure 7A,B. The green vertical lines represent the experimental value of 44.87 ± 0.14 MHz.

The resulting *A*^iso^ distributions of the N- and O-atoms of the HMI nitroxide group are depicted in Figure 12A,B, respectively, and compiled in Table 6 for numerical comparison that also assembles all other model variants tested and analyzed in this work. For the N-atom, the distributions of *A*^iso^ values noticeably shift toward larger values, resulting in an average value of 42.7 MHz that is in favorable agreement with our intrinsic coupled cluster reference based on an explicit solvation value of 40.5 MHz, which in turn is close to the experimental reference of 44.87 ± 0.14 MHz. This implies that both a good solvation model and an accurate electronic structure theory is required to properly compute the average HFCCs of spin probes in an aqueous solution. As EC-RISM calculations with DLPNO-CCSD energetics employ the HF ESP for solute–solvent interactions whereas in the case of revPBE0, consistent polarization with the revPBE0-D3 functional occurs, we further investigated the impact on HFCCs of this inconsistency. To this end, the 400 structures of the DLPNO-CCSD set were treated by revPBE0-D3 and HF electrostatics within EC-RISM, yielding an average *A*^iso^ value of 37.8 MHz, whereas the consistent DFT variant results in an average value of 36.8 MHz (see Supporting Information Figure S4). While apparently the solvent charge distribution derived from the HF density implies a stronger polarity than those generated by the DFT density, the discrepancy originating from inconsistent electrostatics is much smaller than the effect of the improved electronic structure at the CCSD level.

Finally, the difference between thermal averaging of vertically desolvated structures and treatment of the single optimized solution-phase geometry within EC-RISM/DLPNO-CCSD calculations can be determined, also collected in Table 6. The *A*^iso^ value of nitrogen with DLPNO-CCSD at the CPCM minimum energy geometry is 38.1 MHz, which deviates strongly from the respective average of 42.7 MHz, tendencywise in line with DFT results. For the O-atom in HMI, the averaged *A*^iso^ value of −47.8 MHz differs much less from the result for the optimized equilibrium structure (−47.9 MHz).

## 7 Discussion and Conclusions

Calculations of the average isotropic HFCCs of a pH-sensitive EPR probe in solution, HMI in water at ambient conditions, have been performed using a variety of methods, including explicit and EC-RISM solvation at coupled cluster accuracy as provided by the open-shell DLPNO-CCSD method. The explicit solvation configurations were generated from exhaustive AIMD simulations of the EPR probe in water performed using the spin-polarized hybrid functional revPBE0-D3. The canonical ensemble average of the *A*^iso^ parameter at the given thermodynamic conditions was determined from DLPNO-CCSD calculations based on 400 independent solvation configuration snapshots sampled from the AIMD trajectory in the *NVT* ensemble. Furthermore, DFT calculations of this property using 1000 such explicit solvation configurations have also been performed with a commonly used hybrid functional, namely, again revPBE0-D3. The average *A*^iso^ values obtained with different combinations of methods were gauged against the results of a concurrent X-band CW EPR experiment of neutral HMI in water at ambient conditions. The DLPNO-CCSD results in solution are remarkably closer to the experimental value as compared to those obtained when using the hybrid functional. Given that hybrid functionals perform satisfactorily in the gas phase, the current results show that correlated wavefunction-based QC methods in conjunction with proper solvation models are important for quantitative accuracy with EPR parameter calculations in solution. Thus, the current study pushes forward the hitherto considered cutting edge realm of those theoretical methods that are employed to calculate EPR parameters in solution rather than under vacuum (i.e., gas phase).

While we have gone to the limits of what is currently possible in terms of the methodological sophistication, one may critically ask what would be required in order to further push the accuracy limits. In terms of the electronic structure treatment, it would certainly be desirable to incorporate the effects of triple excitations into the response density and hence proceed from DLPNO-CCSD to DLPNO-CCSD(T). For genuine single-reference systems where coupled cluster theory is ideally applicable, the changes brought in by the (T) correction are typically on the order of ∼1 MHz on ^14^N couplings and 1–3 MHz for ^1^H couplings. For example, for NH, the calculated HFCCs of ^14^N and ^1^H change from 17.5 and −63.5 MHz (CCSD) to 17.1 and −62.0 MHz [CCSD(T)] (using the decontracted cc-pVQZ basis set; unpublished data). Thus, given that we have discussed the effects on the order of 1–2 MHz in this study, this additional accuracy would be worthwhile. Beyond including (T) effects will probably prove elusive for some time to come. As we have shown, both adequate solvation modeling and thermal averaging over solute degrees of freedom are equally important in order to achieve high predictive quality. Taking explicit solvation as the benchmark, we demonstrated that the RISM-based approximation of the solvent distribution around the HMI probe outperforms CPCM continuum solvation. Both solvation methodologies, which will be further discussed from a computational perspective in the next paragraph, converge when a sufficiently large explicit solvent environment taken from AIMD is considered in the calculations. Still, even an optimized structure representative for the solvent environment on the CPCM level turned out to be insufficient even within EC-RISM calculations, indicating the strong role of proper thermal averaging of intramolecular degrees of freedom. However, in discussing such small effects, we may also ask how much accuracy is required in order to meaningfully compare the theoretical results to the experiment? In simulations of the EPR spectra, changes on the order of 1 MHz in the HFCCs are barely detectable by either eye or through sensitivity analysis. Only the highest resolution pulse experiments can provide data with an intrinsic accuracy of about 1 MHz, which therefore should probably be considered as “chemical accuracy” in this field. The more readily observable quantity that leads to visible and significant changes in the EPR spectra is the calculated *g*-tensor. However, being a second-order property, this quantity cannot yet be calculated with DLPNO-CCSD and consequently is an important target for method development.

Turning to the computational effort, obtaining coupled cluster level results for systems of the size and complexity studied here only became possible through the recent advances in local correlation theory, and we have demonstrated that these results faithfully mimic the (unobtainable) canonical CCSD results. However, the largest DLPNO-CCSD calculations that include all solvent molecules from AIMD explicitly in the electronic structure treatment are too expensive for production level use or for computing EPR properties along long AIMD trajectories. Hence, we have carefully investigated simplified schemes and found that with a QM/MM embedding approach, the results of the large reference calculations can be faithfully reproduced by only explicitly including solvent molecules up to the second coordination sphere in the quantum region. While this offers essential computational simplifications, the computational effort is still high compared to DFT. Thus, as an alternative representation of the solvent environment, the integral equation-based EC-RISM embedding method has been investigated here in order to further reduce the size of the system that explicitly enters the electronic structure calculation. The average values of the *A*^iso^ parameter calculated for what we call the “vertically desolvated” spin probe molecule configuration (i.e., using the instantaneous HMI configurations but without any solvent molecules from the AIMD trajectory) in conjunction with EC-RISM embedding and explicitly solvated configurations of the probe molecule are in excellent agreement. Thus, EC-RISM embedding provides an alternative, computationally cheaper yet accurate representation of the solvation environment for calculations of EPR observables.

Based on all the results collected above, we advocate the following practical strategy to compute the ensemble averages of *A*^iso^ parameters of EPR probes in aqueous solutions in the sense of the current cutting edge computational protocol: (i) perform sufficiently long AIMD simulations of the spin probe in explicit solvent with a spin-polarized hybrid functional that is capable of a faithful solvation representation of open-shell species in order to generate an uncorrelated set of configuration snapshots, followed by (ii) calculations of *A*^iso^ for all these configurations using the open-shell DLPNO-CCSD correlated wave function method applied to the bare spin probe only, solvated using the EC-RISM embedding technique. However, to give a complete picture, it is also necessary to consider the usability of CPCM and EC-RISM in the current state. Where on the one hand, the continuum models are established in most QC codes and the setting of a suitable dielectric constant for the desired solvent is usually sufficient, employing the EC-RISM model at the present time is not possible without a sophisticated setup procedure. For example, a suitable solvent susceptibility must be elaborately calculated, which depends on several parameters. However, developments are currently underway to facilitate this procedure and thus make EC-RISM easily accessible within QC codes, including the option to compute solvation and reaction-free energies. This approach opens the door to accurately investigate for instance the effects due to local solvation patterns as well as conformational or protonation states of the EPR probe on measured EPR observables.

## Supporting Information Available

Free energy difference calculations between HMI stereoisomers, structural properties of bulk water with the revPBE0-D3 functional, details of force field based molecular dynamics simulations of HMI in water, LJ parameters used in EC-RISM calculations, and distribution plots of *A*^iso^ values of nitrogen and oxygen of the nitroxy group of HMI in explicit water and from solvation models using DLPNO-CCSD and revPBE0-D3 electronic structure theory (PDF)
