# Supplementary material for: A Joint Venture of Ab Initio Molecular Dynamics, Coupled Cluster Electronic Structure Methods, and Liquid-State Theory to Compute Accurate Isotropic Hyperfine Constants of Nitroxide Probes in Water
Source: J Chem Theory Comput. 2021 Sep 13;17(10):6366–86. doi: 10.1021/acs.jctc.1c00582 (PMC8515807; doi:10.1021/acs.jctc.1c00582)
Supplement: Supplementary file 1 — ct1c00582_si_001.pdf [file ct1c00582_si_001.pdf]

Supporting Information

A Joint Venture of Ab Initio Molecular Dynamics, Coupled Cluster Electronic Structure Methods, and Liquid State Theory to Compute Accurate Isotropic Hyperfine Constants of Nitroxide Probes in Water

Bikramjit Sharma,[a] Van Anh Tran,[b] Tim Pongratz,[c] Laura Galazzo,[d] Irina Zhurko,[e] Enrica Bordignon,[d]* Stefan M. Kast,[c]* Frank Neese,[b]* Dominik Marx[a]*

a *Lehrstuhl für Theoretische Chemie, Ruhr-Universität Bochum, 44780 Bochum, Germany*

b *Max-Planck-Institut für Kohlenforschung, Kaiser-Wilhelm-Platz 1, 45470 Mülheim an der Ruhr, Germany*

c *Physikalische Chemie III, Technische Universität Dortmund, Otto-Hahn-Str. 4a, 44227 Dortmund, Germany*

d *Faculty of Chemistry and Biochemistry, Ruhr-Universität Bochum, 44780 Bochum, Germany*

e *Laboratory of Nitrogen Compounds, N.N. Vorozhtsov Novosibirsk Institute of Organic Chemistry, NIOCH SB RAS, 9 Lavrentiev Avenue, 630090, Novosibirsk, Russia*

*Corresponding authors

1 Geometry optimization and Gibbs energies of HMI diastereomers

Table S1. Relative Gibbs energies G_{sol} (in kcal mol^{-1}) and contributions (for EC-RISM:[1] sum of intra-molecular energy E_{sol}, excess chemical potential μ^{ex}, partial molar volume correction c_vV_m with c_v = -0.1022 kcal mol^{-1} Å^{-3}) from calculations on optimized solution structures (Tables S2-S4).

Method / Isomer	E_{sol}	μ^{ex}	c_vV_m	G_{sol} (EC-RISM)	G_{sol} (CPCM)
MP2/6-311+G(d,p)/EC-RISM//B3LYP/6-311+G(d,p)/CPCM					
(3*R*,4*S*)	0	0	0	0	0
(3*R*,4*R*)	5.39	-1.46	0.30	4.23	4.65
MP2/6-311+G(d,p)/EC-RISM//B3LYP/def2-TZVPP/CPCM					
(3*R*,4*S*)	0	0	0	0	0
(3*R*,4*R*)	5.38	-1.42	0.29	4.25	4.90
revPBE0-D3/def2tzvpp/EC-RISM//revPBE0-D3/def2tzvpp/CPCM					
(3*R*,4*S*)	0	0	0	0	0
(3*R*,4*R*)	4.95	-1.63	0.32	3.64	4.44

Table S2. Coordinates (in Å) of optimized solution structures (B3LYP/6-311+G(d,p)/CPCM).

Atom / Isomer	*x*	*y*	*z*	*x*	*y*	*z*
(3*R*,4*S*)				(3*R*,4*R*)		
N	8.252476	8.337704	7.426601	8.037483	8.90737	7.203353
C	7.319935	9.504649	7.635594	7.203373	10.13494	7.310678
N	7.823836	10.492200	6.669654	7.914692	11.01601	6.360682
C	9.289323	10.23990	6.567672	9.340434	10.63951	6.379136
C	9.492319	8.696310	6.681296	9.298843	9.091462	6.438623
O	8.092050	7.214203	8.009302	7.673226	7.789077	7.694933
C	9.509107	7.929554	5.348223	9.149610	8.410110	5.066547
C	10.73106	8.341047	7.512287	10.48055	8.499844	7.211611
H	8.657329	8.186530	4.715495	8.354930	8.874042	4.478577
H	9.476532	6.855247	5.549162	8.908816	7.353909	5.210471
H	10.42671	8.143521	4.794143	10.08452	8.473281	4.505387
H	11.63179	8.679804	6.992909	11.41231	8.719636	6.683294
H	10.80478	7.260596	7.653049	10.38293	7.415113	7.290962
H	10.69975	8.820097	8.493430	10.54571	8.920110	8.217941
C	5.878591	9.045851	7.418225	5.775957	9.851241	6.837061
C	7.486529	10.01918	9.079758	7.210928	10.60304	8.781233
H	5.731616	8.603855	6.432241	5.785080	9.453963	5.820387
H	5.194091	9.888055	7.538599	5.189311	10.77257	6.848138
H	8.490757	10.40685	9.259847	5.290619	9.130518	7.498468
H	7.293350	9.212604	9.791272	8.216148	10.87735	9.109576
H	6.768435	10.82344	9.255861	6.847116	9.794085	9.418564
C	9.980685	10.91229	5.388939	6.551276	11.46342	8.912676
H	9.638396	10.54012	4.422015	10.12790	11.22461	5.210081
H	11.05805	10.73699	5.455200	9.646917	11.00451	4.254008
H	9.817616	11.99278	5.420828	11.14021	10.81177	5.194528
C	7.085344	10.63170	5.408797	10.22031	12.30814	5.307538
H	7.045121	9.729017	4.782137	7.661777	12.44553	6.523109
H	7.539299	11.43054	4.822606	8.119968	12.86920	7.431049
H	6.061427	10.93870	5.624625	6.586739	12.63096	6.560007
H	9.731190	10.66633	7.474085	8.058373	12.98481	5.661688
H	5.633463	8.295706	8.171597	9.814108	10.99061	7.313403

Table S3. Coordinates (in Å) of optimized solution structures (B3LYP/def2-TZVPP/CPCM).

Atom / Isomer	*x*	*y*	*z*	*x*	*y*	*z*
(3*R*,4*S*)			(3*R*,4*R*)			
N	8.256232	8.341695	7.430327	8.038651	8.910431	7.201565
C	7.324564	9.503769	7.632533	7.208654	10.13454	7.310235
N	7.827026	10.48725	6.667828	7.918481	11.01499	6.365774
C	9.288164	10.23748	6.567218	9.33986	10.63933	6.380678
C	9.48926	8.696947	6.682638	9.296449	9.094792	6.440155
O	8.094922	7.220640	8.009366	7.673964	7.793967	7.687767
C	9.505257	7.929457	5.353988	9.149247	8.415227	5.070895
C	10.72743	8.342484	7.508435	10.47465	8.502081	7.210723
H	8.658454	8.187979	4.719426	8.359607	8.880868	4.482023
H	9.465505	6.858282	5.557322	8.90345	7.362685	5.214451
H	10.42305	8.135385	4.802272	10.08325	8.473396	4.512614
H	11.62610	8.675155	6.987045	11.40465	8.713033	6.680968
H	10.79831	7.265055	7.654586	10.37126	7.420783	7.296105
H	10.70049	8.824799	8.485412	10.54469	8.925323	8.212840
C	5.887422	9.043122	7.415080	5.783618	9.853491	6.838199
C	7.484424	10.02014	9.073272	7.213512	10.59971	8.778239
H	5.740025	8.608601	6.428498	5.790725	9.466121	5.820355
H	5.201090	9.879538	7.542339	5.196272	10.77120	6.858238
H	8.484641	10.41095	9.254386	5.301957	9.127418	7.492177
H	7.294214	9.215536	9.784024	8.215219	10.87678	9.106861
H	6.764847	10.81998	9.248005	6.853634	9.790743	9.413493
C	9.978080	10.90801	5.391721	6.552786	11.45602	8.910331
H	9.635943	10.53733	4.426952	10.12388	11.22275	5.213407
H	11.05309	10.73337	5.456771	9.643763	11.00256	4.259742
H	9.814938	11.98605	5.422659	11.13425	10.81177	5.196778
C	7.088366	10.63643	5.414155	10.21539	12.30405	5.309200
H	7.045128	9.741285	4.780618	7.661417	12.43951	6.519974
H	7.539966	11.43715	4.832957	8.112958	12.86962	7.425601
H	6.066878	10.94120	5.632551	6.588071	12.62194	6.552933
H	9.730093	10.66245	7.471834	8.058513	12.9767	5.660365
H	5.647057	8.287866	8.161234	9.817339	10.98892	7.311024

Table S4. Coordinates (in Å) of optimized solution structures (revPBE0-D3/def2-TZVPP/CPCM).

Atom / Isomer	*x*	*y*	*z*	*x*	*y*	*z*
(3*R*,4*S*)			(3*R*,4*R*)			
N	8.256232	8.341695	7.430327	8.038651	8.910431	7.201565
C	7.324564	9.503769	7.632533	7.208654	10.13454	7.310235
N	7.827026	10.48725	6.667828	7.918481	11.01499	6.365774
C	9.288164	10.23748	6.567218	9.33986	10.63933	6.380678
C	9.48926	8.696947	6.682638	9.296449	9.094792	6.440155
O	8.094922	7.220640	8.009366	7.673964	7.793967	7.687767
C	9.505257	7.929457	5.353988	9.149247	8.415227	5.070895
C	10.72743	8.342484	7.508435	10.47465	8.502081	7.210723
H	8.658454	8.187979	4.719426	8.359607	8.880868	4.482023
H	9.465505	6.858282	5.557322	8.90345	7.362685	5.214451
H	10.42305	8.135385	4.802272	10.08325	8.473396	4.512614
H	11.62610	8.675155	6.987045	11.40465	8.713033	6.680968
H	10.79831	7.265055	7.654586	10.37126	7.420783	7.296105
H	10.70049	8.824799	8.485412	10.54469	8.925323	8.212840
C	5.887422	9.043122	7.415080	5.783618	9.853491	6.838199
C	7.484424	10.02014	9.073272	7.213512	10.59971	8.778239
H	5.740025	8.608601	6.428498	5.790725	9.466121	5.820355
H	5.201090	9.879538	7.542339	5.196272	10.77120	6.858238
H	8.484641	10.41095	9.254386	5.301957	9.127418	7.492177
H	7.294214	9.215536	9.784024	8.215219	10.87678	9.106861

Atom / Isomer	*x*	*y*	*z*	*x*	*y*	*z*
(3*R*,4*S*)				(3*R*,4*R*)		
N	8.256232	8.341695	7.430327	8.038651	8.910431	7.201565
H	6.764847	10.81998	9.248005	6.853634	9.790743	9.413493
C	9.978080	10.90801	5.391721	6.552786	11.45602	8.910331
H	9.635943	10.53733	4.426952	10.12388	11.22275	5.213407
H	11.05309	10.73337	5.456771	9.643763	11.00256	4.259742
H	9.814938	11.98605	5.422659	11.13425	10.81177	5.196778
C	7.088366	10.63643	5.414155	10.21539	12.30405	5.309200
H	7.045128	9.741285	4.780618	7.661417	12.43951	6.519974
H	7.539966	11.43715	4.832957	8.112958	12.86962	7.425601
H	6.066878	10.94120	5.632551	6.588071	12.62194	6.552933
H	9.730093	10.66245	7.471834	8.058513	12.9767	5.660365
H	5.647057	8.287866	8.161234	9.817339	10.98892	7.311024

Table S5. Coordinates (in Å) of optimized (3*R*,4*S*) vacuum structure (revPBE0-D3/def2-TZVPP).

Atom	*x*	*y*	*z*
N	7.913225	6.621944	8.039936
C	7.711075	7.468328	9.228306
N	7.704538	8.804437	8.620663
C	8.482493	8.752174	7.384042
C	8.121292	7.379223	6.795154
O	7.7901	5.365998	8.079218
C	6.82233	7.363257	5.989732
C	9.260144	6.766624	5.995318
H	6.530469	6.328791	5.813671
H	6.954408	7.851527	5.024873
H	6.021512	7.864289	6.530558
H	9.453605	7.362457	5.103531
H	9.000124	5.756176	5.686379
H	10.17307	6.718692	6.587418
C	6.363431	7.164709	9.867869
C	8.855246	7.184828	10.20591
H	5.564759	7.328934	9.148182
H	6.198016	7.816114	10.72522
H	6.334358	6.130405	10.20437
H	9.819671	7.458165	9.779399
H	8.867987	6.118233	10.42305
C	8.717537	7.724639	11.14144
H	8.199505	9.930989	6.471426
H	8.570622	10.86009	6.900564
H	7.131331	10.03922	6.29326
C	8.700863	9.797249	5.513098
H	8.06974	9.885717	9.510373
H	7.495546	9.818053	10.4327
H	7.825269	10.84088	9.050864
H	9.137995	9.893318	9.769603
H	9.563533	8.74098	7.605044

2 Details of FFMD for preparing AIMD simulations

Force field simulation of HMI in water

In order to prepare the initial conditions for the AIMD simulation, we carried out force field simulations to pre-equilibrate HMI in water. To this end, we have chosen the TIP4P-2005 water model[2] for these FFMD simulations. For bulk water, this particular force field has been found to perform very well[3]

within the class of simple water models which are both non-polarizable and rigid. Since our goal is to determine the fixed box length to be used in the *NVT* AIMD simulations of HMI in water using FFMD and given that the density of this aqueous solution is largely determined by the density of the solvent itself, the TIP4P-2005 force field is an excellent choice since it reproduces the density of water in a large part of its phase diagram, notably including many ice phases.

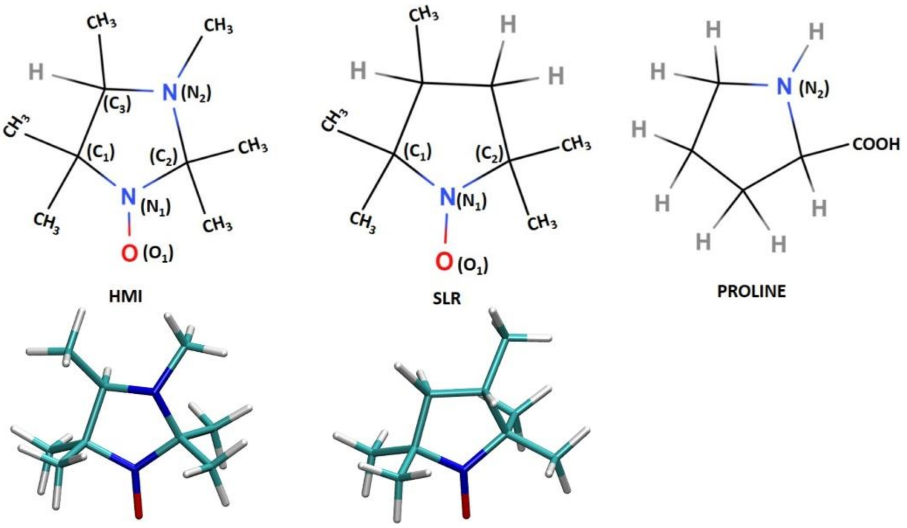

Fig. S1. Comparison of structures of HMI and SLR with proline.

Concerning the force field parameters of HMI, we have borrowed parameters from two structurally similar molecules, namely proline and SLR (Fig. S1). SLR is an EPR probe and differs from HMI in having a carbon atom instead of the other nitrogen site in the ring. Thus, we have adopted parameters of SLR from Ref.[4] and of proline from a recent CHARMM force field[5] to represent similar atoms of HMI (see Table S5 for parameters). With these force field parameters, we have performed FFMD simulations in the *NpT* ensemble for 10 ns using the Gromacs simulation package.[6-9] The pressure and temperature were kept at 1 atm and 300 K using the Parrinello-Rahman[10] barostat and the canonical sampling through a velocity rescaling[11] thermostat, respectively. The average box length of 15.9581 Å obtained from this *NpT* simulation (after equilibration) was taken to be the cubic box parameter for the subsequent runs as follows. A random configuration sampled at this average box length was used to continue the FFMD simulation in the *NVT* ensemble (using this fixed box length) for another 10 ns. The final configuration from this *NVT* run was taken to be the starting configuration for the AIMD simulation. We compare in Fig. S2 the average local solvation structure around the HMI oxygen obtained from FFMD and AIMD (after a sufficiently long trajectory was generated). The deviation of FFMD compared to the AIMD reference is in an acceptable range, thus supporting the use of FFMD for pre-equilibration. However, the differences are substantial enough to not rely on FFMD in order to sample the configuration snapshot ensembles for subsequent single-point property calculations.

Table S6. Atomic charges (in *e*) and atom types of HMI sites adopted for force field molecular dynamics simulations of HMI in water.

HMI atom	SLR atom	Proline atom	Charge
N1	N1	-	0.249
O1	O1	-	-0.433
C1	C1	-	0.245
C2	C2	-	0.267
N2	-	N2	-0.290
C3	-	-	-0.038
Methyl C	-	-	-0.27
Methyl H	-	-	0.09
H bonded to C3	-	-	0.0

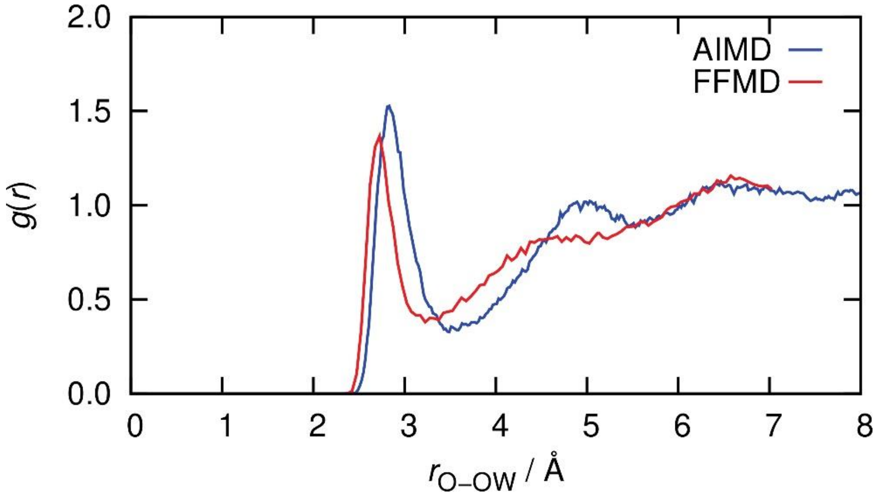

Fig. S2. Comparison of radial distribution functions for water oxygen (OW) with respect to HMI oxygen (O) obtained from AIMD and FFMD.

3 Lennard-Jones parameters for EC-RISM calculations

Table S7. Lennard-Jones parameters *σ* (in Å) and *ε* (in kJ mol^{-1}) of atoms used in EC-RISM calculations. The numbers in parentheses refer to the corresponding GAFF-type atoms.[12]

Atom	*σ*	*ε*
N(3)	3.249999	1.181109
C(3)	3.39967	0.760078
O	2.959922	1.459017
H(C)	2.649533	0.109079
H(1)	2.471353	0.109079

4 Representative snapshots of different QM/MM models

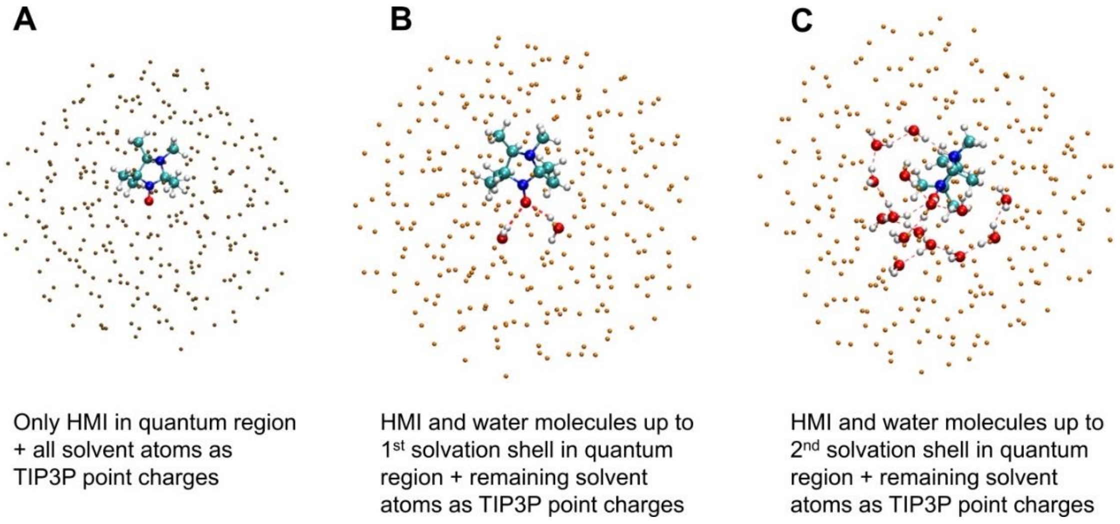

Fig. S3. Representative snapshots of different QM/MM models examined in Section 5.2 of the main text. The dots represent the atoms of all water molecules beyond the QM region which are treated as TIP3P point charges in the MM embedding of the QM subsystem. (A) HMI with no solvation shell considered, (B) HMI+1st solvation shell, and (C) HMI+2nd solvation shell as named in Table 5 of the main text.

5 Comparison of A^{iso} distributions for nitroxy oxygen from DFT

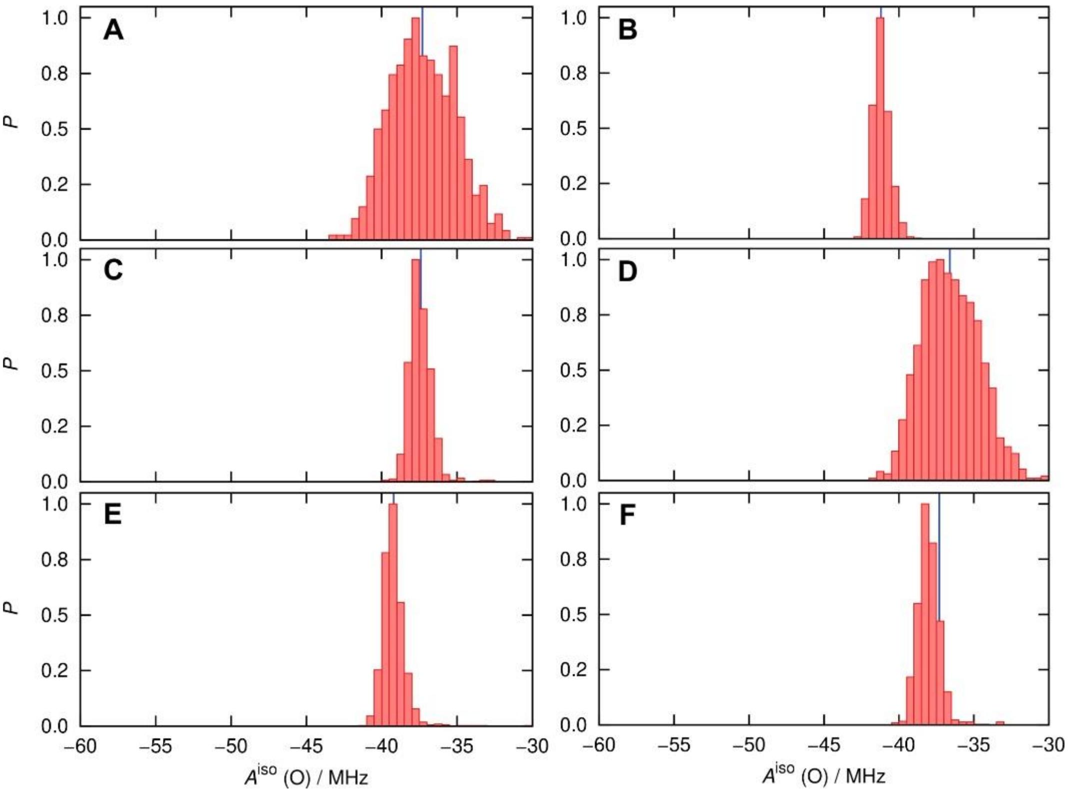

Fig. S4. Probability distributions (normalized by setting the respective maximum bin values to 1) of nitroxy oxygen A^{iso} values calculated with revPBE0-D3 and s-contracted def2-TZVPP basis set; (A) HMI with explicit water, (B) vertically desolvated HMI, (C) vertically desolvated HMI with EC-RISM solvation, (D) vertically desolvated HMI with water molecules up to second solvation shell of HMI oxygen with EC-RISM solvation, (E) vertically desolvated HMI with CPCM solvation, (F) vertically desolvated HMI with water molecules up to second solvation shell of HMI oxygen with CPCM solvation. The blue line in each figure represents the average value of the corresponding distribution.

6 Influence of different EC-RISM electrostatics models

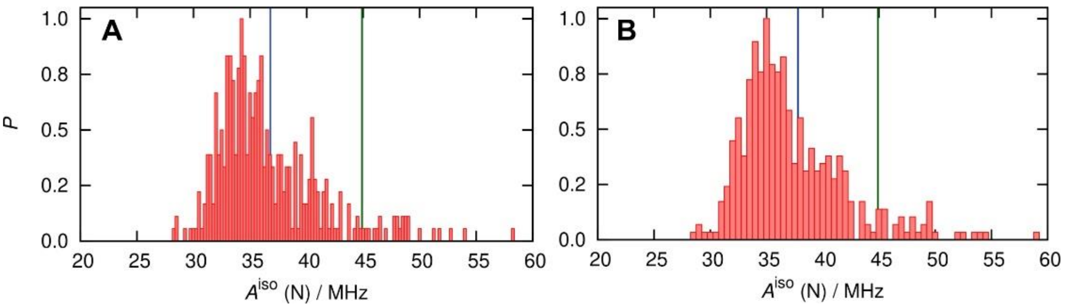

Fig. S5. Probability distributions (normalized by setting the respective maximum bin values to 1) of A^{iso} values of nitroxy nitrogen calculated with revPBE0-D3 and s-contracted def2-TZVPP basis set using the AIMD ensemble of vertically desolvated spin probe configurations; (A) with the consistent DFT electron density to calculate the exact electrostatic potential, (B) with exact electrostatics from the HF electron density, as used within DLPNO-CCSD/def2-TZVPP. The blue line in each figure represents the average value of corresponding distribution. The green vertical lines represent the experimental benchmark value of 44.87 ± 0.14 MHz.

References

1. Tielker, N.; Eberlein, L.; Güssregen, S.; Kast, S. M. The SAMPL6 Challenge on Predicting Aqueous pK_a Values from EC-RISM Theory. *J. Comput. Aid. Mol. Des.* **2018**, *32*, 1151-1163.
2. Abascal, J. L. F.; Vega C. A General Purpose Model for the Condensed Phases of Water: TIP4P/2005. *J. Chem. Phys.* **2005**, *123*, 234505.
3. Vega, C.; Abascal J. L. F.; Simulating Water with Rigid Non-polarizable Models: A General Perspective. *Phys. Chem. Chem. Phys.* **2011**, *13*, 19663–19688.
4. Sezer, D., Freed, J. H., Roux, B. Parametrization, Molecular Dynamics Simulation, and Calculation of Electron Spin Resonance Spectra of a Nitroxide Spin Label on a Polyalanine α-helix. *J. Phys. Chem. B.* **2008**, *112*, 5755–5767.
5. Huang, J.; Rauscher, S.; Nawrocki, G.; Ran, T.; Feig, M.; de Groot B. L. et al. CHARMM36m: An Improved Force Field for Folded and Intrinsically Disordered Proteins. *Nat. Methods* **2017**, *14*, 71–73.
6. Pronk, S.; Páll, S.; Schulz, R.; Larsson, P.; Bjelkmar, P.; Apostolov, R.; et al. GROMACS 4.5: A High-Throughput and Highly Parallel Open Source Molecular Simulation Toolkit. *Bioinformatics* **2013**, *29*, 845–854.
7. van der Spoel, D.; Lindahl, E.; Hess, B.; Groenhof, G.; Mark, A. E.; Berendsen, H. J. C. GROMACS: Fast, Flexible, and Free. *J. Comput. Chem.* **2005**, *26*, 1701–1718.
8. Hess, B.; Kutzner, C.; van der Spoel, D.; Lindahl, E.; GROMACS 4: Algorithms for Highly Efficient, Load-balanced, and Scalable Molecular Simulation. *J. Chem. Theory Comput.* **2008**, *4*, 435–447.
9. Abraham, M. J.; van der Spoel, D.; Lindahl, E.; Hess, B., and the G development team, GROMACS User Manual version 2019, https://www.gromacs.org.
10. Parrinello, M.; Rahman, A. Polymorphic Transitions in Single Crystals: A New Molecular Dynamics Method. *J. Appl. Phys.* **1981**, *52*, 7182–90.
11. Bussi, G.; Donadio, D.; Parrinello, M. Canonical Sampling Through Velocity Rescaling. *J. Chem. Phys.* **2007**, *126*, 014101.
12. Wang, J.; Wolf, R. M.; Caldwell, J. W.; Kollman, P. A.; Case, D. A. Development and Testing of a General Amber Force Field. *J. Comput. Chem.* **2004**, *25*, 1157–1174.
